# Health-Related Quality of Life in Patients With Different Diseases Measured With the EQ-5D-5L: A Systematic Review

**DOI:** 10.3389/fpubh.2021.675523

**Published:** 2021-06-29

**Authors:** Ting Zhou, Haijing Guan, Luying Wang, Yao Zhang, Mingjun Rui, Aixia Ma

**Affiliations:** ^1^School of International Pharmaceutical Business, China Pharmaceutical University, Nanjing, China; ^2^China Center for Health Economic Research, Peking University, Beijing, China

**Keywords:** HRQOL, health utility, EQ-5D-5L, disease, EuroQol

## Abstract

**Background:** The EQ-5D-5L is a generic preference-based questionnaire developed by the EuroQol Group to measure health-related quality of life (HRQoL) in 2005. Since its development, it has been increasingly applied in populations with various diseases and has been found to have good reliability and sensitivity. This study aimed to summarize the health utility elicited from EQ-5D-5L for patients with different diseases in cross-sectional studies worldwide.

**Methods:** Web of Science, MEDLINE, EMBASE, and the Cochrane Library were searched from January 1, 2012, to October 31, 2019. Cross-sectional studies reporting utility values measured with the EQ-5D-5L in patients with any specific disease were eligible. The language was limited to English. Reference lists of the retrieved studies were manually searched to identify more studies that met the inclusion criteria. Methodological quality was assessed with the Agency for Health Research and Quality (AHRQ) checklist. In addition, meta-analyses were performed for utility values of any specific disease reported in three or more studies.

**Results:** In total, 9,400 records were identified, and 98 studies met the inclusion criteria. In the included studies, 50 different diseases and 98,085 patients were analyzed. Thirty-five studies involving seven different diseases were included in meta-analyses. The health utility ranged from 0.31 to 0.99 for diabetes mellitus [meta-analysis random-effect model (REM): 0.83, (95% CI = 0.77–0.90); fixed-effect model (FEM): 0.93 (95% CI = 0.93–0.93)]; from 0.62 to 0.90 for neoplasms [REM: 0.75 (95% CI = 0.68–0.82); FEM: 0.80 (95% CI = 0.78–0.81)]; from 0.56 to 0.85 for cardiovascular disease [REM: 0.77 (95% CI = 0.75–0.79); FEM: 0.76 (95% CI = 0.75–0.76)]; from 0.31 to 0.78 for multiple sclerosis [REM: 0.56 (95% CI = 0.47–0.66); FEM: 0.67 (95% CI = 0.66–0.68)]; from 0.68 to 0.79 for chronic obstructive pulmonary disease [REM: 0.75 (95% CI = 0.71–0.80); FEM: 0.76 (95% CI = 0.75–0.77)] from 0.65 to 0.90 for HIV infection [REM: 0.84 (95% CI = 0.80–0.88); FEM: 0.81 (95% CI = 0.80–0.82)]; from 0.37 to 0.89 for chronic kidney disease [REM: 0.70 (95% CI = 0.48–0.92; FEM: 0.76 (95% CI = 0.74–0.78)].

**Conclusions:** EQ-5D-5L is one of the most widely used preference-based measures of HRQoL in patients with different diseases worldwide. The variation of utility values for the same disease was influenced by the characteristics of patients, the living environment, and the EQ-5D-5L value set.

**Systematic Review Registration**: https://www.crd.york.ac.uk/PROSPERO/, identifier CRD42020158694.

## Background

As a quantitative indicator of health-related quality of life (HRQoL), the health utility reflects people's preference for a given health state. The health utility is measured on a scale from zero to one, where zero represents death and one represents full health (1). The worse the perception of the health status is, the lower the utility value. It can be a negative value when a health state is perceived as being worse than death. There are several preference-based measurement tools for health utility, such as the EuroQol 5 dimensions (EQ-5D) family of instruments (2), the Short Form-6 Dimensions (SF-6D) (3), and the Health Utilities Index (HUI) (4). Health utility can be used as quality-of-life weight to calculate QALYs in cost-utility analysis (CUA). Thus, health utility plays an important role not only in the measurement of HRQoL but also in health economics evaluations (5, 6).

The EQ-5D, developed by the European Quality of Life Group (EuroQol Group), is currently one of the most widely used questionnaires in HRQoL research (7). The original version of the EQ-5D was introduced in 1990 and contains five dimensions: *Mobility, Self-Care, Usual Activities, Pain/Discomfort*, and *Anxiety/Depression* (2). For each dimension, there were three levels to describe the severity, namely, *have no problems, have some problems*, and *have extreme problems*, which could describe 243 different health states (2). However, there may be some issues when using the EQ-5D-3L to detect small changes in mild conditions, and the EQ-5D-3L had obvious ceiling effects (8). Therefore, in 2005, the EuroQol Group developed a new version of the EQ-5D based on the same five dimensions but with five rather than three severity levels (EQ-5D-5L); this instrument could detect 3,125 unique health states (8). Published studies have shown that compared with the EQ-5D-3L, the EQ-5D-5L was significantly more sensitive, with reduced ceiling effects (9, 10).

To derive health utility from the responses on the EQ-5D instruments, country-specific value sets need to be estimated (11). Since 2016, more than 20 countries and regions have published standard EQ-5D-5L value sets (Europe: 9; Asia: 9; Americas: 3; Africa: 1) (12). In 2012, before any standard EQ-5D-5L value set was established, van Hout et al. (13) developed a *crosswalk* project to map the EQ-5D-5L to the EQ-5D-3L, enabling researchers to obtain a crosswalk value set for the EQ-5D-5L based on published EQ-5D-3L standard value sets. Besides that, the psychometric properties of the EQ-5D-5L have been validated in both general and disease populations (12).

In recent years, with the availability of the EQ-5D-5L value sets, an increasing number of studies have used the EQ-5D-5L to measure the HRQoL of patients with different diseases and perform economic evaluations to support health decision-making (14, 15). At present, a comprehensive review of these studies is lacking. For HRQoL measured with EQ-5D-5L, cross-sectional studies mainly focus on the current health status of the patients while randomized controlled trials (RCTs) pay attention to the effects of different interventions on health outcomes. This study focuses on the use of the EQ-5D-5L to explore the variation in health utility in patients in different conditions, provide information to perform CUAs, and inform health policies.

## Method

### Search Strategy and Study Inclusion Criteria

This systematic review and meta-analysis was performed in accordance with Preferred Reporting Items for Systematic Reviews and Meta-Analyses (PRISMA) guidelines (16). The protocol was registered on PROSPERO with ID *CRD42020158694* (https://www.crd.york.ac.uk/PROSPERO/). Literature searches were conducted in Medline *via* Ovid, Embase *via* Ovid, The Cochrane Library, and Web of Science from January 2012 to October 2019 with combinations of the following search terms: “quality of life,” “QoL,” “HRQoL,” “HRQL,” “EQ-5D,” “EQ-5D-5L,” “five level,” “EuroQol,” “five dimensions,” “randomized controlled trial,” “RCT,” and “diseases” (details in Supplementary Table 1).

According to the selection criteria, all studies were original cross-sectional studies reporting EQ-5D-5L utilities for any specific disease with or without comorbidities and using country-specific value sets or the crosswalk method (mapping from EQ-5D-3L). Due to the lack of EQ-5D-5L standard value sets in many countries, the crosswalk method is the most important value set to calculate utility measured by EQ-5D-5L. In addition, the crosswalk method is recommended by the National Institute for Health and Care Excellence (NICE) to perform CUA when EQ-5D-5L is used to measure health outcomes in England. Therefore, it is useful and necessary to include these articles in this review. Studies reported that multiple utility values using value sets from different countries in the same published article were also included. The language of publication was limited to English. This review excluded reviews, protocols, or abstracts; studies focused on the general population; longitudinal studies or effects evaluation studies of different interventions; studies that reported only synthetic utilities of multiple diseases, non-EQ-5D-5L utilities, or no utilities; and studies unrelated to HRQoL.

### Data Collection and Quality Assessment

After removing duplicates, title and abstract screening was conducted by two authors independently. Following the application of the selection criteria, all eligible studies with full-texts were read, and the relevant references were checked manually. Two researchers independently collected the data using a predesigned data extraction table, including author, publication year, country or region, sample size, disease type, mean age, health utility, EQ-5D VAS score, proportions with problems in the five dimensions, value set, and administration method (i.e., face-to-face, telephone survey). When there was any discrepancy between the two researchers, it was resolved by discussion.

Quality assessment was conducted with the 11-item cross-sectional research checklist developed by the Agency for Healthcare Research and Quality (AHRQ) (17). According to the description in the study and the AHRQ checklist, the reviewer selects one of three options (“Yes,” “No,” and “Unclear”) for each item. “Yes” was assigned one point, while “No” or “Unclear” was assigned zero points. The quality level of each study was determined by summing all the item scores. For each assessed study, 0–3 points indicated low quality, 4–7 points indicated moderate quality, and 8–11 points indicated high quality.

### Statistical Analysis

This review involved the analysis of the range of mean health utility values of the overall sample (or subgroups when there is no overall utility value reported) among different studies and value sets used in each study for a specific disease with or without comorbidities. In addition, this study reports the ranges in mean EQ-VAS scores and responses on each dimension of the EQ-5D-5L.

Meta-analysis was performed to synthesize utility data when three or more studies reported utility values and standard error/deviation for a specific disease. For any study that reported multiple utility values for the same sample using different EQ-5D-5L value sets, the average value or the utility calculated by using a local country-specific value set was applied in meta-analysis. Heterogeneity was assessed with the *I*^2^ statistic. Random-effect (DerSimonian–Laird estimator method) and fixed-effect (inverse variance method) models were both used to calculate the pooled utility for a specific disease. Sensitivity analysis was conducted by removing EQ-5D-5L utility values derived from crosswalk value sets. All analyses were performed with R (version 4.0.5).

## Results

**Figure 1:**
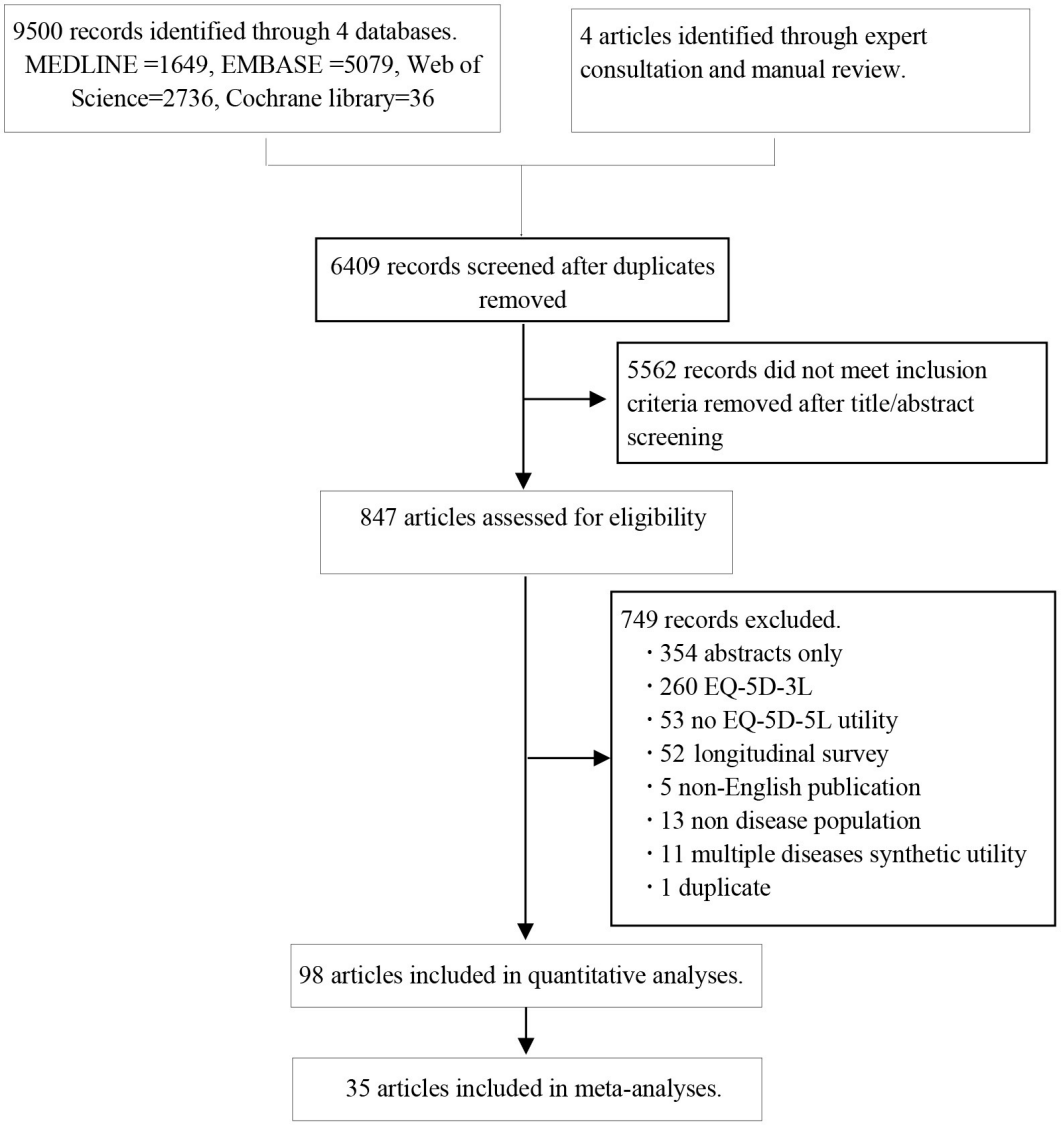
Flow diagram of article selection for inclusion.

A total of 9,500 articles were identified from the four databases, and four additional studies were obtained from the manual search. After eliminating duplicates, 6,409 documents were screened to assess eligibility, of which 98 articles (15, 16, 18–113) were finally included in qualitative analyses and 35 studies were included in meta-analyses (Figure 1). Those 98 articles involved 98,085 patients. The included studies were published between January 2006 and March 2018 (Table 1). Except for three studies (29, 39, 79) that only included male patients and one study (96) that only included female patients, the rest of the studies included patients of both sexes. Twenty studies did not report the mode of administration. Of the remaining 78 studies, 47.4% involved the face-to-face administration of the survey, 47.4% involved self-administered surveys, and 5.2% involved telephone surveys. The AHRQ checklist scores ranged from four to nine points, the median was six points, and the mode was five points (details in Supplementary Table 2). There were no low-quality studies; 87 studies and 11 studies were of moderate and high quality, respectively. The data about the distributions of EQ-5D-5L are summarized in Supplementary Table 3.

**Table 1:** Basic characteristics of the included studies.

**Author year**	**Country/region**	**Survey time**	**Sample size**	**Male (%)**	**Diseases**	**Age (SD)**	**AHQR scores**
Natasya et al. 2018 (14)	Indonesia	October to December 2017	108	31.5	Diabetes mellitus (type 2)	-	5
Sothornwit et al. 2018 (15)	Thailand	January 2014 to September 2016	254	47.0	Diabetes mellitus	63.2 (12.1)	6
Pan et al. 2018 (18)	China	2015	722	43.1	Diabetes without diabetic retinopathy	67.9 (8.2)	5
			56	44.6	Diabetes with unilateral retinopathy	68.9 (7.4)	
			102	51.0	Diabetes with bilateral retinopathy	65.3 (8.7)	
Lamu et al. 2018 (19)	Australia, Canada, Germany, Norway, UK and USA	2012	924	58.7	Diabetes	55.9 (12.6)	7
Adibe et al. 2018 (20)	Nigeria	-	147	44.9	Diabetes mellitus (type 2)	-	5
Arifin et al. 2019 (21)	Indonesia	November 2015 to October 2017	907	57.0	Diabetes mellitus (type 2)	59.3 (9.7)	6
Schmitt et al. 2018 (22)	Germany	September 2015 to August 2016	606	45.2	Diabetes mellitus	50 (15)	7
Collado et al. 2015 (23)	Spain	July 2011 to June 2012	1,857	45.3	Diabetes mellitus	≥18	6
Khatib et al. 2018 (24)	Palestine	November 2016 to June 2017	141	52.5	Diabetes mellitus (type 2)	60.3	8
Zyoud et al. 2015 (25)	Palestine	June 2013 to October 2013	385	44.9	Diabetes mellitus (type 2)	59.3 (11.2)	5
Xu et al. 2017 (26)	China	July to December 2014	1,721	-	Heart disease	≥18	5
			4,528	-	Hypertension	≥18	
			2,326	-	Diabetes	≥18	
			267	-	Cancer	≥18	
Pan et al. 2016 (27)	China	March 2014 to June 2014	289	30.5	Diabetes mellitus (type 2)	64.9 (9.1)	7
Huang et al. 2018 (28)	China	December 2016 to April 2017	300	65.0	Colorectal cancer	59	7
Gavin et al. 2016 (29)	Republic of Ireland	2012	1,431	100.0	Prostate cancer early stage	64.9 (7.6)	7
			407	100.0	Prostate cancer late stage	64.9 (7.6)	
	Northern Ireland	2012	269	100.0	Prostate cancer early stage	64.9 (7.6)	
			282	100.0	Prostate cancer late stage	64.9 (7.6)	
Lloyd et al. 2015 (30)	UK	-	50	100.0	Prostate cancer asymptomatic/mildly symptomatic	71.8 (8.8)	5
			50	100.0	Prostate cancer currently receiving chemotherapy	69.8 (11.9)	
			12	100.0	Prostate cancer symptomatic before chemotherapy	59.9 (15.2)	
			46	100.0	Prostate cancer post chemotherapy	68.4 (9.24)	
Philipp-Dormston et al. 2018 (31)	Germany	October 2015 to February 2016	869	61.3	Actinic keratosis	74	8
			578	61.3	Basal cell carcinoma	74	
			204	61.3	Squamous cell carcinoma	74	
Noel et al. 2015 (32)	Canada	August 2014 to October 2014	100	75.0	Squamous cell carcinoma	61	5
Mastboom et al. 2018 (33)	Netherlands	December 2016 to May 2017	69	20.3	Localized tenosynovial giant cell tumor	41	6
			230	22.2	Diffuse tenosynovial giant cell tumor	41	
Zhang et al. 2017 (34)	Australia	2015	231	36.8	Progressive-onset multiple sclerosis	61.8 (9.6)	7
			1,514	18.4	Relapse-onset multiple sclerosis	53.5 (11.0)	
Algahtani et al. 2017 (35)	Saudi Arabia	June 2016 to April 2017	292	30.8	Multiple sclerosis	35.9 (10.3)	7
Fogarty et al. 2012 (36)	Ireland	-	214	33.6	Multiple sclerosis	47.6 (12.8)	6
Carney et al. 2018 (37)	Ireland	Spring of 2015	541	28.7	Multiple sclerosis	47	7
Nohara et al. 2017 (38)	Japan	2016	96	38.5	Multiple sclerosis	47.5 (14.2)	7
Barin et al. 2018 (39)	Switzerland	June 2016 to September 2017	855	27.3	Multiple sclerosis	48.0 (38.6)	8
Buanes et al. 2015 (40)	Norway	October 2012	30	80.0	Cardiac arrest	62	5
Berg et al. 2017 (41)	Denmark	April 2013 to April 2014	7,179	73	Ischemic heart disease	65.5	9
			4,322	65	Arrhythmia	63.6	
			987	73	Heart failure	65.4	
			115	47	Congenital heart disease	43.9	
			204	75	Infectious heart disease	59.4	
			975	66	Heart valve disease	71.2	
			136	74	Heart transplant	51.2	
			321	61	Other diagnoses of heart disease	61.4	
			2,473	53	Observation for heart disease	61.5	
Squire et al. 2017 (42)	UK	January to May 2015	191	73.0	Heart failure	70	6
Meroño et al. 2017 (43)	Spain	November 2012 to October 2015	139	66.0	Iron deficiency in acute coronary syndrome	67 (15)	9
			105	83.0	Acute coronary syndrome non-iron deficiency	61 (12)	
Tran et al. 2018 (44)	Vietnam	July to December 2016	600	41.5	Cardiovascular disease	57.2	5
Wang et al. 2018 (45)	China		234	43.0	Atrial fibrillation	60	5
De Smedt et al. 2016 (46)	24 European countries	2012 to 2013	7,449	76.1	Stable coronary disease	64	5
Garcia-Gordillo et al. 2017 (47)	Spain	July 2011 and June 2012	1,130	48.7	COPD	15-102	5
Igarashi et al. 2018 (48)	Japan	-	71	84.5	COPD age <65 years	60.5 (5.3)	6
			151	95.4	COPD age ≥ 65 years	75.2 (5.9)	
Lin et al. 2014 (49)	USA	2006 to 2010	670	58.0	COPD	68.5 (10.4)	6
Nolan et al. 2016 (50)	UK	April 2012 to October 2014	616	59.7	COPD	70.4 (9.3)	8
Keaei et al. 2016 (51)	Colombia	May to June 2014	138	77.5	HIV/AIDS	46.4 (11.4)	7
Dang et al. 2018 (52)	Vietnam	January to August 2013	1,133	58.7	HIV-positive	35.5 (6.9)	7
Tran et al. 2012 (53)	Vietnam	2012	1,016	63.8	HIV	35.4 (7.0)	6
Van Duin et al. 2017 (54)	Columbia		100	77.0	HIV with comorbidities	48.0 (11.2)	5
			38	21.1	HIV without comorbidities	42.2 (11.1)	
Yang et al. 2015 (55)	Singapore	June 2012 to May 2013.	150	51.3	End-stage renal disease	60.1 (11.6)	6
Hiragi et al. 2019 (56)	Japan	July 2015 to March 2017	67	62.7	Chronic kidney disease (TR)	49.8 (13.1)	4
			65	53.8	Chronic kidney disease (TRC)	49.4 (11.6)	
Zyoud et al. 2016 (57)	Palestine	June 2014 to January 2015	267	52.1	End-stage renal disease	53.3 (16.2)	8
Al-Jabi et al. 2015 (58)	Palestine	July 2012 and October 2012	410	48.0	Hypertension	58.4 (10.7)	8
van der Linde et al. 2017 (59)	Netherland	January 2006 to December 2014.	101	77.2	Midshaft clavicular fractures	44.5 (13.6)	7
Larsen et al. 2015 (60)	Denmark	Autumn 2013 to spring 2014	48	77.1	Femoral shaft fracture	38.0 (19.4)	6
Kim et al. 2018 (61)	Korea	August 2014 to February 2017.	59	11.9	Osteoporotic vertebral compression fracture	73.5 (6.2)	6
Chevreul et al. 2016 (62)	France	September 2012 to May 2013	38	45.0	Prader–Willi syndrome	17.4 (12.2)	4
López-Bastida et al. 2016 (63)	UK	September 2011 to April 2013	26	-	Prader–Willi syndrome	13.7 (8.5)	5
	Sweden		10	-	Prader–Willi syndrome	16.0 (9.2)	
	Spain		61	-	Prader–Willi syndrome	14.9 (10.8)	
	Germany		52	-	Prader–Willi syndrome	10.8 (9.5)	
	Italy		48	-	Prader–Willi syndrome	13.6 (9.6)	
	France		51	-	Prader–Willi syndrome	17.4 (12.2)	
Vaizey et al. 2014 (64)	UK	October 2011 to March 2012	100	55.0	Ulcerative colitis remission	47.5	5
			31	48.4	Ulcerative colitis mild	48	
			42	40.5	Ulcerative colitis moderate/severe	40.5	
Gibson et al. 2014 (65)	Australia	July to October 2011	94	47.4	Ulcerative colitis remission	47.8 (12.7)	5
			29	47.4	Ulcerative colitis mild	47.8 (12.7)	
			52	47.4	Ulcerative colitis moderate/severe	47.8 (12.7)	
Yfantopoulos et al. 2017 (66)	Greece	December 2012 to March 2013	396	60.1	Psoriasis	52.0 (16.5)	5
Zhao et al. 2017 (67)	China	May 2014 to February 2015	350	69.7	Psoriasis	39	7
Choi et al. 2018 (68)	Korea	January to December 2017	105	76.0	Ankylosing spondylitis	39	5
Chiowchanwisawaki et al. 2019 (69)	Thailand	May 2012 to March 2016	119	61.3	Ankylosing spondylitis	40.4 (11.6)	5
Alvarado-Bolaños et al. 2015 (70)	Mexico	-	585	54.4	Parkinson's disease	62.9 (12.3)	4
Garcia-Gordillo et al. 2014 (71)	Spain	May 1 to July 15, 2012	133	71.4	Parkinson's disease	64.3 (9.7)	6
Lee et al. 2015 (72)	South Korea	July to December 2013	625	32.5	Overactive bladder	63.5 (12.0)	6
Lloyd et al. 2017 (73)	UK	2014	249	54.6	Idiopathic overactive bladder	57.3/58.1	6
Nordenfelt et al. 2017 (74)	Sweden	May and October 2016.	64	40.6	Hereditary angioedema	51	6
Nordenfelt et al. 2014 (75)	Sweden	June 2011	103	47.6	Hereditary angioedema	41/44	4
Whitehurst et al. 2016 (76)	Canada	March to June 2013	364	62.9	Spinal cord injury	50.40 (13.2)	8
Engel et al. 2018 (77)	Canada	March to June 2013	364	62.9	Spinal cord injury	50.4 (13.2)	3
Buckner et al. 2017 (78)	USA	September to November 2015	299	71.0	Hemophilia B	29	5
Kempton et al. 2018 (79)	USA	October 2013 to October 2014	381	100.0	Hemophilia	34	7
Arraras et al. 2018 (80)	Spain	May 2015 to June 2016	61	66.0	Schizophrenia and schizoaffective disorder	37.9 (10.5)	7
Kitic et al. 2018 (81)	Serbia	-	153	54.9	Schizophrenia	50.8 (10.1)	4
Tennvall et al. 2015 (82)	Denmark	May to June in 2012	312	51.9	Actinic keratosis	71 (11.0)	7
Gray et al. 2018 (83)	Australia, Canada, Germany, Norway, the United Kingdom, and the United States	2012	852	37.7	Asthma	43.0 (15.0)	4
Hernandez et al. 2018 (84)	French		222	38.7	Asthma	30.3 (6.7)	8
Wong et al. 2018 (85)	UK	March 2014 to January 2017	990	19.7	Autoimmune hepatitis	58	7
Cook et al. 2019 (86)	Canada, Germany, UK, and USA	-	166	49.4	Non-alcoholic steatohepatitis	52.0 (11.8)	5
van Dongen-Leunis et al. 2016 (87)	Netherlands	2012	111	52.3	Acute leukemia	51.0 (13.4)	6
Hendriksz et al. 2014 (88)	Brazil, Colombia, Germany, Spain, Turkey, UK	June 2012 to April 2013	25	-	Morquio A syndrome adults	≥18	5
			33	-	Morquio A syndrome children	5-17	
Andersson et al. 2016 (89)	France, Germany, Spain, USA	February to May 2013	1,104	59.1	Nocturia	65.1	8
Mealy et al. 2019 (90)	USA	October 6, 2014	21	90.5	Neuromyelitis optica spectrum disorder	42.8 (10.6)	5
Nikiphorou et al. 2018 (91)	Multinational	-	3,370	66.0	Spondyloarthritis	42.9 (13.7)	5
Van Assche et al. 2016 (92)	11 European countries	-	250	58.8	Ulcerative colitis	46.6 (16.3)	6
Mijnarends et al. 2016 (93)	Dutch	May 2013 to February 2014	53	52.8	Sarcopenia	80.4 (7.1)	7
Tran et al. 2018 (94)	Vietnam	September to November 2017.	223	51.1	Dengue fever	31.6 (12.4)	7
Chevreul et al. 2015 (95)	France	September 2012 to May 2013	82	42.7	Cystic fibrosis	28.6 (8.1)	5
Collado-Mateo et al. 2017 (96)	Spain	October 2014 to October 2015	192	0.0	Fibromyalgia	53.8 (10.0)	5
Chevreul et al. 2015 (97)	France	September 2012 to May 2013	95	87.4	Fragile X syndrome	19.4 (13.1)	5
Juul-Kristensen et al. 2017 (98)	Denmark	January to June 2015	300	24.3	Generalized joint hypermobility	48	6
Bewick et al. 2018 (99)	UK	January 2013 to January 2014	52	51.0	Rhinosinusitis	55	6
Forestier-Zhang et al. 2016 (100)	UK	September 2014 to March 2016.	43	23.0	Osteogenesis imperfecta	40.4 (14.4)	6
			42	31.0	Fibrous dysplasia	44.3 (14.5)	
			24	21.0	X-linked hypophosphatemia	46.3 (16.3)	
Katchamart et al. 2019 (101)	Thailand	September 2016 to March 2018	464	14.9	Rheumatoid arthritis	59.2 (11.4)	5
Román Ivorra et al. 2019 (102)	Spain	October 2015 to March 2016	190	7.9	Systemic lupus erythematosus	47.2 (13.4)	5
Aguirre et al. 2016 (103)	UK	-	272	39.0	Dementia	82.6 (8.1)	5
Wong et al. 2017 (104)	China	August to October 2015	227	25.1	Adolescent idiopathic scoliosis	15.5 (3.8)	5
Christensen et al. 2016 (105)	Norway	June 27 to July 3, 2014	188	13.9	Opioid-induced constipation	≥18	5
Vo et al. 2018 (106)	France, Germany, Italy, Spain, UK	2016	218	20.6	Migraine	43.3 (13.5)	6
Voormolen et al. 2019 (107)	UK, the Netherlands and Italy	June 29th to July 31st 2017	11,759	49.7	Post-concussion syndrome	44	5
Lim et al. 2017 (108)	Singapore	2013	100	59.0	Stoma	64 (9.7)	5
Villoro et al. 2016 (109)	Spain	2011–2012	14,691	28.3	Chronic depression	48.3 (11.0)	7
Vermaire et al. 2016 (110)	Netherlands	July 2013 to June 2015	76	42.1	severe dental anxiety	42.6 (11.9)	6
Lane et al. 2017 (111)	UK	January 2011 and July 2012	330	47.0	Symptomatic varicose vein	52	6
Rencz et al. 2018 (112)	Hungary	October 2016 to September 2017	206	54.9	Crohn's disease	34.7 (10.5)	7
Chevreul et al. 2015 (113)	France	September 2012 to May 2013	147	9.5	Systemic sclerosis	53.8 (11.7)	7

*SD, standard deviation; AHQR, agency for health research and quality; COPD, chronic obstructive pulmonary disease; HIV, human immunodeficiency virus; AIDS, acquired immunodeficiency syndrome; TRC, transplant recipient candidate; TR, transplant recipient*.

In this review, health utility values derived from the EQ-5D-5L were reported for 50 different diseases. Among these, diabetes mellitus, neoplasms, multiple sclerosis, cardiovascular disease, chronic obstructive pneumonia disease (COPD), human immunodeficiency virus (HIV) infection, chronic kidney disease, and fracture were reported in three or more studies and meta-analyses were performed for these diseases (fracture was not included in meta-analysis, because only two of the studies reported standard error/deviation). The sensitivity analysis results (remove all the utility values derived from the crosswalk value set) are presented in Supplementary Figure 1.

### Diabetes Mellitus

For patients with diabetes mellitus (Table 2), 12 studies reported health utility values ranging from 0.31 to 0.99 (14, 15, 18–27). The Chinese standard EQ-5D-5L value set (18) and Crosswalk UK value set (24) were used to derive the utility values in the studies that reported the highest value and lowest value, respectively. The former focused on diabetes patients without diabetic retinopathy with a mean disease duration of 10.3 years and a mean age of 67.9 years (18), while the latter involved patients with severe comorbidities on hemodialysis, with a mean age of 60.3 years (24). Additionally, Lamu et al. (19) used eight country value sets (England, the Netherlands, Spain, Canada, Uruguay, China, Japan, and Korea) to analyze 924 diabetic patients from six countries. The results showed that the utility value calculated with the Uruguay value set was the highest at 0.880, while the lowest, 0.735, was derived with the value set from the Netherlands. The EQ-5D VAS scores were reported to range from 50.9 to 72.6 in six studies (14, 20, 22–25). Among the five dimensions of the EQ-5D-5L, pain/discomfort was the dimension with the most reported problems. The prevalence of diabetes comorbidities ranged from 55 to 100%, which was one of the most important factors negatively affecting the HRQoL of patients.

**Table 2:** HRQoL in patients with different diseases measured by the EQ-5D-5L.

	**Diseases**	**Health Utility**			**VAS scores**		**Have any problem in 5 dimensions (%)**					**Administration**
		**Mean**	**SD**	**Value set^※^**	**Mean**	**SD**	**MO**	**SC**	**UA**	**PA**	**AD**	
**Diabetes mellitus**												
Natasya et al. 2018 (14)	Diabetes mellitus (type 2)	0.74	0.23	Indonesia	65.5	16.0	44.4	16.6	27.8	64.8	58.3	-
Sothornwit et al. 2018 (15)	Diabetes mellitus	0.80	0.25	Thailand	-	-	-	-	-	-	-	Face-to-face
Pan et al. 2018 (18)	Diabetes without diabetic retinopathy	0.99	0.05	China	-	-	7.1	1.1	0.7	7.9	3.2	Telephone
	Diabetes with unilateral retinopathy	0.97	0.08	China	-	-	12.5	5.4	5.4	16.1	8.9	Telephone
	Diabetes with bilateral retinopathy	0.97	0.15	China	-	-	7.8	3.9	5.9	9.8	5.9	Telephone
Lamu et al. 2018 (19)	Diabetes mellitus	0.79	0.22	England	-	-	-	-	-	-	-	Self-administered
	Diabetes mellitus	0.74	0.26	Dutch	-	-	-	-	-	-	-	Self-administered
	Diabetes mellitus	0.76	0.21	Spain	-	-	-	-	-	-	-	Self-administered
	Diabetes mellitus	0.78	0.19	Canada	-	-	-	-	-	-	-	Self-administered
	Diabetes mellitus	0.88	0.14	Uruguay	-	-	-	-	-	-	-	Self-administered
	Diabetes mellitus	0.76	0.25	China	-	-	-	-	-	-	-	Self-administered
	Diabetes mellitus	0.77	0.19	Japan	-	-	-	-	-	-	-	Self-administered
	Diabetes mellitus	0.78	0.17	Korea	-	-	-	-	-	-	-	Self-administered
Adibe et al. 2018 (20)	Diabetes mellitus (type 2)	0.72	0.13	-	72.6	10.5	61.2	32.0	62.6	83.0	71.4	Face-to-face
Arifn et al. 2019 (21)	Diabetes mellitus (type 2)	0.77	-	Indonesia	-	-	37.0	12.0	23.0	61.0	34.0	Self-administered
Schmitt et al. 2018 (22)	Diabetes mellitus	0.80	0.20	Crosswalk (Germany)	66.0	20.0	-	-	-	-	-	-
Collado et al. 2015 (23)	Diabetes mellitus	0.74	0.32	Crosswalk (Spain)	61.1	20.5	46.8	23.6	37.5	54.4	29.4	Face-to-face
Khatib et al. 2018 (24)	Diabetes mellitus (type 2)	0.31	-	Crosswalk (UK)	50.9	22.4	-	-	-	-	-	Face-to-face
Zyoud et al. 2015 (25)	Diabetes mellitus (type 2)	0.70	0.20	-	63.7	19.2	-	-	-	-	-	Face-to-face
Xu et al. 2017 (26)	Diabetes mellitus	0.84	0.23	Hong Kong	-	-	-	-	-	-	-	Telephone survey
Pan et al. 2016 (27)	Diabetes mellitus (type 2)	0.88	0.14	Crosswalk (China)	-	-	-	-	-	-	-	Self-administered
**Neoplasms**												
Huang et al. 2018 (28)	Colorectal cancer	0.62	0.37	China	-	-	46.3	49.0	53.3	60.3	59.3	Face-to-face.
Gavin et al. 2016 (29)	Prostate cancer late stage (RoI)	0.80	-	Crosswalk (UK)	-	-	-	-	-	-	-	Self-administered
	Prostate cancer late stage (NI)	0.70	-	Crosswalk (UK)	-	-	-	-	-	-	-	Self-administered
	Prostate cancer early stage (RoI)	0.90	-	Crosswalk (UK)	-	-	-	-	-	-	-	Self-administered
	Prostate cancer early stage (NI)	0.80	-	Crosswalk (UK)	-	-	-	-	-	-	-	Self-administered
Lloyd et al. 2015 (30)	Prostate cancer asymptomatic/mildly symptomatic	0.83	0.13	Crosswalk^Δ^	77.5	12.6	-	-	-	-	-	Self-administered
	Prostate cancer currently receiving chemotherapy	0.69	0.22	Crosswalk^Δ^	67.4	14.3	-	-	-	-	-	Self-administered
	Prostate cancer symptomatic before chemotherapy	0.63	0.17	Crosswalk^Δ^	56.2	16.7	-	-	-	-	-	Self-administered
	Prostate cancer post chemotherapy	0.70	0.18	Crosswalk^Δ^	66.0	17.9	-	-	-	-	-	Self-administered
Philipp-Dormston et al. 2018 (31)	Basal cell carcinoma	0.87^*^	-	Dutch	-	-	-	-	-	-	-	-
	Squamous cell carcinoma	0.84	-	Dutch	-	-	-	-	-	-	-	-
Noel et al. 2015 (32)	Squamous cell carcinoma	0.82	0.18	-	76.0	19.0	-	-	-	-	-	Face-to-face
Mastboom et al. 2018 (33)	Diffuse tenosynovial giant cell tumor	0.72	-	Crosswalk (US)	-	-	-	-	-	-	-	Self-administered
	Localized tenosynovial giant cell tumor	0.76	-	Crosswalk (US)	-	-	-	-	-	-	-	Self-administered
Xu et al. 2017 (26)	Cancer	0.84	0.22	Hong Kong	-	-	-	-	-	-	-	Telephone survey
**Multiple sclerosis**												
Zhang et al. 2017 (34)	Relapse-onset multiple sclerosis	0.73	0.22	-	-	-	-	-	-	-	-	Self-administered
	Progressive-onset multiple sclerosis	0.54	0.27	-	-	-	-	-	-	-	-	Self-administered
Algahtani et al. 2017 (35)	Multiple sclerosis	0.31	0.51	Crosswalk (UK)	73.9	23.4	72.9	60.3	68.2	71.9	73.6	Face-to-face
Fogarty et al. 2012 (36)	Multiple sclerosis	0.59	0.33	Crosswalk^Δ^	65.0	22.4	70.1	36.2	70.6	67.3	54.2	Face-to-face
Carney et al. 2018 (37)	Multiple sclerosis	0.59	0.29	Crosswalk (UK)	63.3	21.7	-	-	-	-	-	Self-administered
Nohara et al. 2017 (38)	Multiple sclerosis	0.68	0.19	-	58.3	27.0	-	-	-	-	-	Self-administered
Barin et al. 2018 (39)	Multiple sclerosis	0.78	-	Crosswalk (France)	78.0	-	-	-	-	-	-	Face-to-face
**Cardiovascular disease**												
Buanes et al. 2015 (40)	Cardiac arrest	0.85	-	-	70.6	-	-	-	-	-	-	Self-completed
Berg et al. 2017 (41)	Ischemic heart disease	0.76	0.16	Crosswalk^Δ^	68.6	19.7	-	-	-	-	-	Self-administered
	Arrhythmia	0.70	0.16	Crosswalk^Δ^	72.2	19.6	-	-	-	-	-	Self-administered
	Heart failure	0.73	0.16	Crosswalk^Δ^	61.4	19.5	-	-	-	-	-	Self-administered
	Congenital heart disease	0.77	0.16	Crosswalk^Δ^	69.9	19.7	-	-	-	-	-	Self-administered
	Infectious heart disease	0.73	0.16	Crosswalk^Δ^	68.4	19.6	-	-	-	-	-	Self-administered
	Heart valve disease	0.74	0.16	Crosswalk^Δ^	66.1	19.7	-	-	-	-	-	Self-administered
	Heart transplant	0.82	0.16	Crosswalk^Δ^	76.0	19.6	-	-	-	-	-	Self-administered
	Other diagnoses of heart disease	0.73	0.16	Crosswalk^Δ^	65.3	19.5	-	-	-	-	-	Self-administered
	Observation for heart disease	0.76	0.16	Crosswalk^Δ^	70.5	19.6	-	-	-	-	-	Self-administered
Squire et al. 2017 (42)	Heart failure	0.60	0.25	UK	63.0	20.0	-	-	-	-	-	Self-administered
Merono et al. 2017 (43)	Iron deficiency in acute coronary syndrome	0.76	0.25	-	66.0	16.0	52.0	20.0	49.0	50.0	61.0	Self-administered
	Acute coronary syndrome non-iron deficiency	0.84	0.16	-	72.0	17.0	29.0	12.0	33.0	49.0	52.0	Self-administered
Tran et al. 2018 (44)	Cardiovascular disease	0.82	0.21	Crosswalk^Δ^	77.8	13.6	24.8	19.8	22.7	38.8	35.2	Face-to-face
Wang et al. 2018 (45)	Atrial fibrillation	0.56	-	China	-	-	-	-	-	-	-	Face-to-face
Xu et al. 2017 (26)	Heart disease	0.84	0.24	Hong Kong	-	-	-	-	-	-	-	Telephone survey
De Smedt et al. 2016 (46)	Stable coronary disease	0.78	0.20	Crosswalk^Δ^	67.1	21.4	-	-	-	-	-	-
**COPD**												
Garcia-Gordillo et al. 2017 (47)	COPD	0.74	0.31	Crosswalk^Δ^	60.5	21.9	45.4	22.2	37.5	57.1	34.9	Face-to-face.
Igarashi et al. 2018 (48)	COPD age ≥ 65 years	0.77	0.18	Japan	69.2	18.7	56.3	26.5	46.7	37.7	35.1	Self-administered
	COPD age <65 years	0.79	0.22	Japan	70.5	23.8	43.7	23.9	43.7	30.0	38.6	Self-administered
Lin et al. 2014 (49)	COPD	0.79	0.15	Crosswalk^Δ^	70.6	19.6	63.6	19.5	54.8	61.9	36.3	-
Nolan et al. 2016 (50)	COPD	0.68	0.24	UK	61.0	20.6	-	-	-	-	-	-
**HIV infection**												
Keaei et al. 2016 (51)	HIV/AIDS	0.85	0.21	Crosswalk (Spain)	84.4	14.3	18.8	8.7	15.9	38.4	40.6	Face-to-face
Dang et al. 2018 (52)	HIV-positive	0.80	0.20	-	68.8	17.3	20.5	9.7	16.6	37.7	44.9	Face-to-face
Tran et al. 2012 (53)	HIV	0.65	-	Crosswalk (Thailand)	70.3	-	45.1	20.2	35.4	58.2	72.5	Face-to-face
Van Duin et al. 2017 (54)	HIV with comorbidities	0.84	0.22	Crosswalk (Spain)	84.4	16.1	-	-	-	-	-	-
	HIV without comorbidities	0.90	0.19	Crosswalk (Spain)	88.6	10.4	-	-	-	-	-	-
**Chronic kidney disease**												
Yang et al. 2015 (55)	End-stage renal disease	0.68	0.36	Crosswalk (UK)	-	-	-	-	-	-	-	Face-to-face
Hiragi et al. 2019 (56)	Chronic kidney disease (TRC)	0.89	0.15	Japan	-	-	-	-	-	-	-	Face-to-face
	Chronic kidney disease (TR)	0.85	0.16	Japan	-	-	-	-	-	-	-	Face-to-face
Zyoud et al. 2016 (57)	End-stage renal disease	0.37	0.44	Crosswalk (UK)	59.4	45.4	27.3	54.7	37.5	25.5	35.2	Face-to-face
**Hypertension**												
Al-Jabi et al. 2015 (58)	Hypertension	0.80	0.16	Crosswalk (UK)	74.1	15.6	-	-	-	-	-	Face-to-face
Xu et al. 2017 (26)	Hypertension	0.85	0.22	Hong Kong	-	-	-	-	-	-	-	Telephone survey
**Fractures**												
Van der Linde et al. 2017 (59)	Midshaft clavicular fractures	0.88	0.14	-	77.2	26.8	-	-	-	-	-	Self-administered
Larsen et al. 2015 (60)	Femoral shaft fracture	0.80	-	Crosswalk (Denmark)	80.3	-	-	-	-	-	-	-
Kim et al. 2018 (61)	Osteoporotic vertebral compression fracture	0.56	0.24	-	-	-	-	-	-	-	-	-
**Prader–Willi syndrome**												
Chevreul et al. 2016 (62)	Prader–Willi syndrome	0.44	0.33	Crosswalk^Δ^	59.5	17.7	-	-	-	-	-	Face-to-face
López-Bastida et al. 2016 (63)	Prader–Willi syndrome (UK)	0.48	0.22	-	56.9	19.7	-	-	-	-	-	Self-administered
	Prader–Willi syndrome (Sweden)	0.63	0.10	-	51.3	10.3	-	-	-	-	-	Self-administered
	Prader–Willi syndrome (Spain)	0.60	0.78	-	62.6	20.5	-	-	-	-	-	Self-administered
	Prader–Willi syndrome (Italy)	0.40	0.29	-	56.2	19.7	-	-	-	-	-	Self-administered
	Prader–Willi syndrome (Germany)	0.81	0.14	-	60.7	26.4	-	-	-	-	-	Self-administered
	Prader–Willi syndrome (France)	0.41	0.34	-	56.5	17.7	-	-	-	-	-	Self-administered
**Ulcerative colitis**												
Vaizey et al. 2014 (64)	Ulcerative colitis remission	0.86	0.15	Crosswalk^Δ^	-	-	-	-	-	-	-	Face-to-face
Gibson et al. 2014 (65)	Ulcerative colitis remission	0.81	0.18	-	-	-	-	-	-	-	-	-
Vaizey et al. 2014 (64)	Ulcerative colitis moderate/severe	0.66	0.24	Crosswalk^Δ^	-	-	-	-	-	-	-	Face-to-face
Gibson et al. 2014 (65)	Ulcerative colitis moderate/severe	0.68	0.19	-	-	-	-	-	-	-	-	-
Vaizey et al. 2014 (64)	Ulcerative colitis mild	0.77	0.11	Crosswalk^Δ^	-	-	-	-	-	-	-	Face-to-face
Gibson et al. 2014 (65)	Ulcerative colitis mild	0.78	0.18	-	-	-	-	-	-	-	-	-
**Psoriasis**												
Yfantopoulos et al. 2017 (66)	Psoriasis	0.74	0.23	Crosswalk^Δ^	74.7	18.1	18.4	9.8	15.7	33.6	78.0	Self-administered
Zhao et al. 2017 (67)	Psoriasis	0.90	0.10	China	72.7	15.7	-	-	-	-	-	Face-to-face
	Psoriasis	0.86	0.10	Japan	72.7	15.7	-	-	-	-	-	Face-to-face
	Psoriasis	0.90	0.09	UK	72.7	15.7	-	-	-	-	-	Face-to-face
**Ankylosing spondylitis**												
Choi et al. 2018 (68)	Ankylosing spondylitis	0.69^*^	-	Japan	-	-	-	-	-	-	-	-
Chiowchanwisawakit et al. 2019 (69)	Ankylosing spondylitis	0.75	0.20	Thailand	68.8	18.8	77.3	37.0	68.9	93.3	54.6	Face-to-face
**Actinic keratosis**												
Tennvall et al. 2015 (82)	Actinic keratosis	0.88	0.14	Crosswalk (Denmark)	79.3	18.9	21.0	7.0	18.0	39.0	22.0	-
Philipp-Dormston et al. 2018 (31)	Actinic keratosis	0.89^*^	-	Dutch	-	-	-	-	-	-	-	-
**Parkinson's disease**												
Alvarado-Bolaños et al. 2015 (70)	Parkinson's disease	0.71	0.20	Crosswalk (US)	73.8	18.7	-	-	-	-	-	Self-administered
Garcia-Gordillo et al. 2014 (71)	Parkinson's disease	0.59	0.26	Crosswalk (Spain)	57.6	19.7	75.9	60.2	75.9	75.9	66.2	-
**Overactive bladder**												
Lee et al. 2015 (72)	Overactive bladder	0.79	0.20	Crosswalk (UK)	-	-	-	-	-	-	-	Self-administered
Lloyd et al. 2017 (73)	Idiopathic overactive bladder	0.73	0.26	-	68.2	21.6	-	-	-	-	-	Face-to-face
**Hereditary angioedema**												
Nordenfelt et al. 2017 (74)	Hereditary angioedema	0.84^*^	-	UK	-	-	-	-	-	-	-	Self-administered
Nordenfelt et al. 2014 (75)	Hereditary angioedema	0.83	0.21	Crosswalk^Δ^	-	-	-	-	-	-	-	Self-administered
**Spinal cord injury**												
Whitehurst et al. 2016 (76)	Spinal cord injury	0.49	0.20	Canada	-	-	97.0	67.0	80.0	93.0	57.0	Self-administered
Engel et al. 2018 (77)	Spinal cord injury	0.49	0.20	Canada	-	-	-	-	-	-	-	Self-administered
**Schizophrenia**												
Arraras et al. 2018 (80)	Schizophrenia and schizoaffective disorder	0.80	0.21	-	58.8	19.6	-	-	-	-	-	Face-to-face
Kitic et al. 2018 (81)	Schizophrenia	0.86	0.13	-	50.0	13.8	-	-	-	-	-	Face-to-face
**Hemophilia**												
Buckner et al. 2017 (78)	Hemophilia B	0.67	-	Crosswalk (US)	54.4	-	78.0	75.0	87.0	93.0	81.0	Self-administered
Kempton et al. 2018 (79)	Hemophilia	0.77	-	Crosswalk (US)	65.6	-	61.4	18.9	53.2	76.1	43.4	-
**Asthma**												
Gray et al. 2018 (83)	Asthma	0.84	0.17	UK	-	-	-	-	-	-	-	Self-administered
Hernandez et al. 2018 (84)	Asthma	0.83	0.17	Crosswalk (French)	77.3	16.5	-	-	-	-	-	Telephonic interviews
**Hepatitis**												
Wong et al. 2018 (85)	Autoimmune hepatitis	0.89^*^	-	UK	80.0	-	-	-	-	-	-	-
Cook et al. 2019 (86)	Non-alcoholic steatohepatitis	0.81	0.17	-	67.2	18.9	-	-	-	-	-	Telephone survey
**Other diseases**												
van Dongen-Leunis et al. 2016 (87)	Acute leukemia	0.81	0.22	Dutch	-	-	-	-	-	-	-	Self-administered
	Acute leukemia	0.85	0.18	UK	-	-	-	-	-	-	-	Self-administered
Hendriksz et al. 2014 (88)	MAS use wheelchair when needed (children)	0.66	-	-	-	-	-	-	-	-	-	Self-administered
	MAS use wheelchair when needed (adult)	0.58	-	-	-	-	-	-	-	-	-	Self-administered
	MAS don't need wheelchair (children)	0.53	-	-	-	-	-	-	-	-	-	Self-administered
	MAS don't need wheelchair (adult)	0.85	-	-	-	-	-	-	-	-	-	Self-administered
	MAS always use wheelchair (children)	−0.18	-	-	-	-	-	-	-	-	-	Self-administered
	MAS use wheelchair (adult)	0.06	-	-	-	-	-	-	-	-	-	Self-administered
Andersson et al. 2016 (89)	Nocturia	0.78	-	UK	-	-	-	-	-	-	-	Self-administered
Mealy et al. 2019 (90)	Neuromyelitis optica spectrum disorder	0.74	0.16	Crosswalk^Δ^	-	-	66.7	33.3	61.9	76.2	71.4	Face-to-face.
Nikiphorou et al. 2018 (91)	Spondyloarthritis	0.60	0.30	-	-	-	-	-	-	-	-	-
Van Assche et al. 2016 (92)	Ulcerative colitis	0.77	0.19	-	70.5	19.1	-	-	-	-	-	-
Mijnarends et al. 2016 (93)	Sarcopenia	0.78	0.19	Crosswalk^Δ^	72.0	16.0	-	-	-	-	-	Face-to-face
Tran et al. 2018 (94)	Dengue fever	0.66	0.24	Crosswalk^Δ^	-	-	62.3	71.8	64.6	32.3	64.1	Face-to-face
Chevreul et al. 2015 (95)	Cystic fibrosis	0.67	0.25	Crosswalk (French)	65.6	20.0	-	-	-	-	-	Self-administered
Collado-Mateo et al. 2017 (96)	Fibromyalgia	0.49	0.26	Crosswalk (Spain)	-	-	-	-	-	-	-	Face-to-face
Chevreul et al. 2015 (97)	Fragile X syndrome	0.49	0.24	Crosswalk^Δ^	70.0	-	-	-	-	-	-	Self-administered
Juul-Kristensen et al. 2017 (98)	Generalized joint hypermobility	0.82^*^	-	Crosswalk^Δ^	80^*^	-	-	-	-	-	-	Self-administered
Bewick et al. 2018 (99)	Rhinosinusitis	0.75	0.23	UK	73.4	-	30.8	9.6	39.5	67.3	42.3	Face-to-face
Forestier-Zhang et al. 2016 (100)	Fibrous dysplasia	0.66	0.29	UK	64.1	23.0	57.0	38.0	67.0	98.0	62.0	Self-administered
	X-linked hypophosphatemia	0.65	0.29	UK	60.8	26.9	87.0	50.0	75.0	92.0	58.0	Self-administered
	Osteogenesis imperfecta	0.66	0.28	UK	69.4	21.4	81.0	39.0	65.0	93.0	60.0	Self-administered
Katchamart et al. 2019 (101)	Rheumatoid arthritis	0.87	0.13	-	79.4	17.0	51.5	16.8	35.3	70.5	38.8	-
Román Ivorral et al. 2019 (102)	Systemic lupus erythematosus	0.74	0.25	-	65.7	23.5	-	-	-	-	-	Face-to-face
Aguirre et al. 2016 (103)	Dementia	0.78	0.23	-	64.1	20.5	-	-	-	-	-	-
Wong et al. 2017 (104)	Adolescent idiopathic scoliosis	0.93	0.11	Crosswalk (China)	-	-	-	-	-	-	-	Self-administered
Christensen et al. 2016 (105)	Opioid-induced constipation	0.59	0.27	-	60.7	22.6	-	-	-	-	-	Self-administered
Vo et al. 2018 (106)	Migraine	0.68	-	-	-	-	-	-	-	-	-	Self-administered
Voormolen et al. 2019 (107)	Post-concussion syndrome	0.81	0.23	Dutch	74.7	19.6	-	-	-	-	-	Self-administered
Lim et al. 2017 (108)	Stoma	0.80	0.16	Crosswalk (UK)	76.0	8.7	-	-	-	-	-	-
Villoro et al. 2016 (109)	Chronic depression	0.74	0.28	Spain	-	-	30.1	13.3	28.6	57.4	73.5	Face-to-face
Vermaire et al. 2016 (110)	Severe dental anxiety	0.70^*^	-	Crosswalk^Δ^	-	-	-	-	-	-	-	Face-to-face
Lane et al. 2017 (111)	Symptomatic varicose vein	0.74^*^	-	-	80^*^	-	-	-	-	-	-	Face-to-face
Rencz et al. 2018 (112)	Crohn's disease	0.87	0.12	UK	72.7	19.7	4.8	1.5	11.6	17.8	5.9	Self-administered
Chevreul et al. 2015 (113)	Systemic sclerosis	0.49	0.25	Crosswalk (France)	59.0	18.0	-	-	-	-	-	Face-to-face

*SD, standard deviation; VAS, visual analogue scale; HRQoL, health-related quality of life; EQ-5D-5L, 5 level version of EuroQol 5-Dimensions; MO, mobility; SC, self-care; UA, usual activities; PD, pain/discomfort; AD, anxiety/depression; RoI, the Republic of Ireland; NI, Northern Ireland; COPD, chronic obstructive pulmonary disease; HIV, human immunodeficiency virus; AIDS, acquired immunodeficiency syndrome; TRC, transplant recipient candidate; TR, transplant recipient; MAS, Morquio A syndrome*.

※ *Crosswalk method is using the EQ-5D-3L standard value set to calculate EQ-5D-5L utility values*.

* *Only reported median value*.

Δ *The country of crosswalk method not reported*.

**Figure 2:**
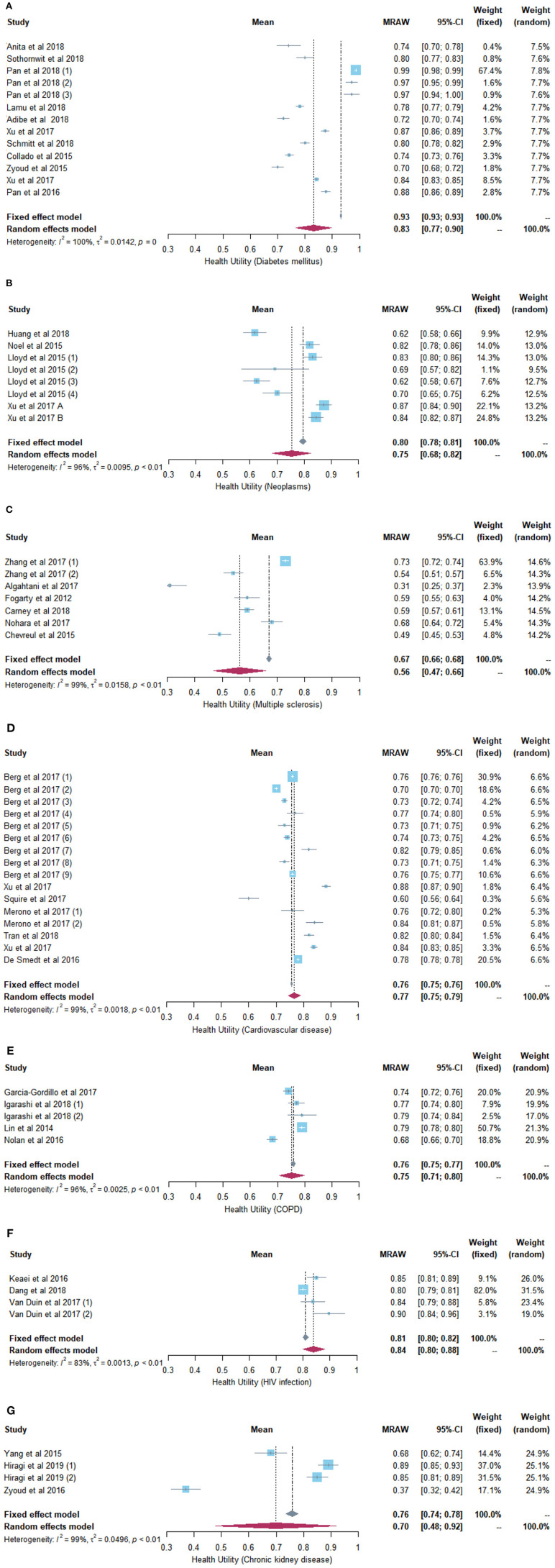
**(A)** Forest plot of the health utility of patients with diabetes mellitus. **(B)** Forest plot of the health utility of patients with neoplasms. **(C)** Forest plot of the health utility of patients with multiple sclerosis. **(D)** Forest plot of the health utility of patients with cardiovascular diseases. **(E)** Forest plot of the health utility of patients with chronic obstructive pneumonia disease. **(F)** Forest plot of the health utility of patients with human immunodeficiency virus infection. **(G)** Forest plot of the health utility of patients with chronic kidney disease.

The meta-analytic utility estimate of diabetes mellitus was 0.83 (95% confidence interval (CI) = 0.77–0.90, heterogeneity *I*^2^ = 100%, *P* = 0.00) using the random-effect model, and it was 0.93 (95% CI = 0.93–0.93) using the fixed-effect model. The results are presented in Figure 2A.

### Neoplasms

Seven studies reported health utility values for cancer patients ranging from 0.62 to 0.90 (26, 28–33). The highest utility value was in early-stage prostate cancer patients using the crosswalk UK value set (29), while the lowest value was in colorectal cancer patients, 49.7% of whom had stage III–IV disease, applying the China value set (28). The EQ-5D VAS scores ranged from 56.2 to 77.5 in two studies (30, 32). The decrease in health utility in cancer patients was mainly due to problems related to the pain/discomfort dimension of the EQ-5D-5L. As the cancer progressed, the health utility value decreased.

The pooled utility value of cancer patients was 0.75 (95% CI = 0.68–0.82, heterogeneity *I*^2^ = 96%, *P* <0.01) using the random-effect model, and it was 0.80 (95% CI = 0.78–0.81) using the fixed-effect model (Figure 2B).

### Multiple Sclerosis

The health utility ranged from 0.31 to 0.78 for multiple sclerosis patients in six studies (34–39). The upper and lower utility values were generated with the crosswalk France value set (35) and the crosswalk UK value set (39), respectively. The study with the highest value (39) reported a shorter disease duration (9 vs. 15 years) than the study with the lowest utility value (35). In addition, the former had a higher proportion of relapsing–remitting multiple sclerosis patients than the latter (71.5 vs. 52.8%). EQ-5D VAS scores ranged from 58.3 to 78.0 in five studies (35–39). Pain/discomfort and usual activities were the dimensions with the most reported problems among multiple sclerosis patients.

The meta-analytic utility estimate of multiple sclerosis patients was 0.56 (95% CI = 0.47–0.66, heterogeneity *I*^2^ = 99%, *P* <0.01) using the random-effect model, and it was 0.67 (95% CI = 0.66–0.68) using the fixed-effect model (Figure 2C).

### Cardiovascular Disease

For cardiovascular disease patients, the health utility values ranged from 0.56 to 0.85 in eight studies (26, 40–46). The lowest value was derived from the Chinese value set (45), while the study with the highest value did not report the value set used (40). In the study with the highest utility value (40), all patients were evaluated 4 years after cardiac arrest, and the proportion of men was 80%. In the study with the lowest value, the patients had atrial fibrillation; 43% of them were men, and 23% had diabetes mellitus (45). Berg et al. (41) compared utility values among nine subgroups of patients with different cardiovascular diseases. Among these subgroups, heart transplant patients had the highest value, which was 0.82, while arrhythmia patients had the lowest value, which was 0.70. The EQ-5D VAS scores ranged from 61.4 to 77.8 in six studies (26, 40–44). Anxiety/depression and pain/discomfort were the dimensions with the most reported problems among cardiovascular disease patients.

The pooled utility value of cardiovascular disease patients was 0.77 (95% CI = 0.75–0.79, heterogeneity *I*^2^ = 99%, *P* <0.01) using the random-effect model, and it was 0.76 (95% CI = 0.75–0.76) using the fixed-effect model (Figure 2D).

### COPD

For patients with COPD, the health utility values ranged from 0.68 to 0.79 in four studies (47–50). The crosswalk US value set and UK standard EQ-5D-5L value set were used in the studies that reported the highest utility value (49) and the lowest value (50), respectively. The mean age of COPD patients in the study reporting the lowest utility was 70.4 years, and the mean predicted forced expiratory volume in 1 s (FEV1) was 49.8% (50). Meanwhile, the patients in the study with the highest value had a younger mean age (68.5 years old) and a better predicted FEV1 (49). The EQ-5D VAS scores ranged from 60.5 to 70.6 in four studies (47–50). Mobility was the dimension with the most problems affecting the HRQoL of COPD patients based on EQ-5D-5L. In addition, as the predicted FEV1 decreased, the health utility value in COPD patients decreased.

The synthesized utility value of COPD patients was 0.75 (95% CI = 0.71–0.80, heterogeneity *I*^2^ = 96%, *P* <0.01) using the random-effect model, and it was 0.76 (95% CI = 0.75–0.77) using the fixed-effect model (Figure 2E).

### HIV Infection

The health utility values of patients infected with HIV ranged from 0.65 to 0.90 in four studies (51–54), and both extreme values were derived with a crosswalk value set [Thailand (53) and Spain (54)]. The study (54) with the highest utility value involved patients in relatively good condition and without any comorbidities, while the study (53), with the lowest value focused on patients who had symptomatic HIV infections. The EQ-5D VAS scores ranged from 68.8 to 88.6 in four studies (51–54). The decrease in utility in HIV-infected patients was mainly due to problems related to the anxiety/depression dimension of the EQ-5D-5L.

The pooled utility value of patients infected with HIV was 0.84 (95% CI = 0.80–0.88, heterogeneity *I*^2^ = 83%, *P* <0.01) using the random-effect model, and it was 0.81 (95% CI = 0.80–0.82) using the fixed-effect model (Figure 2F).

### Chronic Kidney Disease

For chronic kidney disease patients, the health utility values ranged from 0.37 to 0.89 in three studies (55–57). The Japan value set and crosswalk UK value set were used to calculate the highest utility value (56) and the lowest value (57), respectively. The mean age of chronic kidney disease patients in the study reporting the highest value was 49.8 years old, and all of them had received kidney transplants (56), while those in the study reporting the lowest value were 59.4 years old, and 33.7% of them had been on dialysis for 4 years or longer (57). One study (57) reported that the EQ-5D VAS score was 59.4. Among the five dimensions, self-care was the dimension with the most reported problems among chronic kidney disease patients.

The meta-analytic utility estimate of chronic kidney disease was 0.70 (95% CI = 0.48–0.92, heterogeneity *I*^2^ = 99%, *P* <0.01) using the random-effect model, and it was 0.76 (95% CI = 0.74–0.78) using the fixed-effect model (Figure 2G).

### Fracture

The health utility values of patients with fractures ranged from 0.56 to 0.88 in the three studies (59–61). However, neither of the studies that reported the maximum and minimum values described the value sets used (59, 61). The patients in the study reporting the highest value (59) had midshaft clavicular fractures and a much younger mean age (44.5 vs. 73.5 years old) than the osteoporotic vertebral compression fracture patients in the study reporting the lowest value (61). Two studies reported EQ-5D VAS scores of 80.3 (60) and 77.2 (59). No information was available for the dimensions that contributed the most to the HRQoL of fracture patients.

### Other Diseases

For Prader–Willi syndrome, hypertension, ulcerative colitis, ankylosing spondylitis, psoriasis, actinic keratosis, Parkinson's disease, overactive bladder, hereditary angioedema, spinal cord injury, schizophrenia, hemophilia, asthma, and hepatitis, only two studies reported the health utility values for patients with each disease. For the remaining 29 diseases (87–113), the HRQoL and utility values were only reported by one study each. Patients with adolescent idiopathic scoliosis had the highest utility value of 0.93 (104), while children with Morquio A syndrome, who must use wheelchairs, had the lowest value of −0.18 (88).

Furthermore, two studies compared utility values calculated with different country-specific value sets in the same sample (67, 87). For patients with psoriasis living in central South China (67), value sets for Japan, China, and the UK were used separately to obtain the EQ-5D-5L utility values, and the results were 0.86, 0.90, and 0.90, respectively. van Dongen-Leunis et al. (87) used two EQ-5D-5L country-specific value sets to calculate the health utility of acute leukemia patients, and the value derived from the Dutch value set (0.81) was lower than that derived from the UK value set (0.85). The rest of the studies all used a single value set. Compared with other dimensions, pain/discomfort was the dimension with the most problems reported by patients in most of the studies.

## Discussion

In this study, we reviewed the health utility values in patients with different diseases according to the EQ-5D-5L in cross-sectional surveys. We found that the EQ-5D-5L has been widely applied in populations with specific diseases, including various chronic non-communicable diseases, such as diabetes mellitus, neoplasms, multiple sclerosis, and cardiovascular disease, and infectious diseases, such as HIV and Dengue fever. The health utility values for a specific disease measured by the EQ-5D-5L differed based on patient characteristics, survey location, the use of country-specific value sets, and other factors. Meta-analyses were performed to synthesized utility data of any specific disease reported in three or more studies.

Health utility measures the preference of people for a given health state and reflects their status with regard to quality of life (1). Sex is one of the factors that affect health utilities (47). There are differences in the perception of health status between males and females, and in most of the included studies that reported sex-specific utilities, men had better HRQoL as measured by the EQ-5D-5L than women. For instance, the utility value was 0.80 for men with COPD and 0.69 for women with COPD, and the proportion of men who reported having problems on all five dimensions was lower than the proportion of women (47). In addition, health utility values decreased as the age of patients increased due to the deterioration of physical function and reduced disease tolerance. Among patients with COPD, for example, the utility value for patients under 65 years of age (0.77) was lower than that for patients who were 65 years old and older (0.79) (48).

In general, the severity of disease is reflected by the magnitude of the health utility value. The variation in values measured by EQ-5D-5L for the same disease under different conditions reflects its discriminative ability. As the disease progresses, the utility value decreases. Alvarado-Bolaños et al. (70) used Hoehn and Yahr staging to categorize Parkinson's disease patients into groups with mild, moderate, and severe disease, and the utility values were 0.77, 0.65, and 0.47, respectively. In addition, the number of comorbidities and the different types of comorbidities substantially affect the HRQoL of patents. Patients who have comorbidities usually report a lower utility value than those without comorbidities. Van Duin et al. (54) reported that the utility value was 0.90 in patients with HIV infections who did not have any comorbidities; however, it was reduced to 0.84 when patients had comorbid diseases. In Al-Jabi's study (58), for hypertension patients with one, two, and three or more comorbidities, the utility values were 0.81, 0.73, and 0.66, respectively.

Various living environments result in different lifestyles, which may influence HRQoL and health utility. Zyoud et al. (57) reported that among patients with end-stage renal disease in Palestine, those living in villages had a higher mean utility value than those living in cities (0.44 vs. 0.29). In another study (44), among patients with cardiovascular disease, the utility value was a little bit higher for those living in urban Vietnam than those in rural areas (0.82 vs. 0.81).

To calculate health utility, the target patients' responses to the EQ-5D-5L and a country-specific value set are needed. The health preferences of patients living in different countries are affected by their social environment, living standards, and health system. Therefore, the EQ-5D-5L value sets estimated based on residents' preferences for health states vary across countries or regions. Different results can be observed in the same sample when various country value sets are used to calculate health utility values. In the same sample of patients with acute leukemia, van Dongen-Leunis et al. (87) reported that the value obtained with the Dutch value set was higher than that obtained with the UK value set. In countries where the EQ-5D-5L utility value set has been estimated, it is more appropriate to use the local value set. Before any standard country-specific EQ-5D-5L value set was published, the crosswalk method developed by van Hout et al. (13) in 2012 was an alternative means of calculating health utility measured by EQ-5D-5L. For cost-utility analyses performed in England, the NICE recommends the use of the crosswalk method to obtain EQ-5D-5L utility values and calculate quality-adjusted life-years (QALYs) because there are some concerns about the current standard value set published by Devlin et al. (114). In this review, a crosswalk value set was used in half of the studies to calculate utility values due to the lack of a local standard EQ-5D-5L value set when the survey was conducted. Therefore, the crosswalk value set is still important for researchers to calculate health utility.

The heterogeneity of health utility derived from different studies for any specific disease is significant. Although, this may lead to some issues of the direct comparison among these studies, the trend of variation and the influence factors of health utility can be observed. In addition, to perform CUA, different sources of health utilities are need to be identified and applied in the model (1). The summarization and review of health utility for different diseases are helpful and useful.

There are some limitations of this study. Among the 50 different diseases analyzed in this review, nearly half of them were only discussed in one study each. The included studies were limited to those published in English. In addition, some of the studies did not describe the value set used. This review focused on health utility measured by the EQ-5D-5L in cross-sectional studies, and the comparison of different utility-based instruments (i.e., SF-6D, HUI) in populations with specific diseases needs further exploration.

A deeper understanding of the HRQoL and health utility of patients with different diseases facilitates the provision of a more appropriate range of services for disease management and treatment. In addition, health utility is used for HRQoL weighting when calculating QALYs. QALY is used as the outcome measure in CUA and plays an important role in health technology assessments (12). The summarization of health utility from various sources provides information to perform CUA which could inform health decision making and the reasonable allocation of health resources.

## Data Availability Statement

The original contributions presented in the study are included in the article/Supplementary Material, further inquiries can be directed to the corresponding author/s.

## Author Contributions

TZ, HG, and AM designed this study protocol. HG, AM, and TZ conceived the literature strategies. LW and MR reviewed the title/abstract independently. TZ and LW performed the original study review. TZ, HG, and YZ extracted and analyzed the data from included studies. TZ and MR assessed the methodological quality with AHRQ checklists. TZ and YZ contributed to the writing of the manuscript. All the authors approved the final version of this systematic review.

## Conflict of Interest

The authors declare that the research was conducted in the absence of any commercial or financial relationships that could be construed as a potential conflict of interest.
