# Supplementary material for: Health-Related Quality of Life in Patients With Different Diseases Measured With the EQ-5D-5L: A Systematic Review
Source: Front Public Health. 2021 Jun 29;9:675523. doi: 10.3389/fpubh.2021.675523 (PMC8275935; doi:10.3389/fpubh.2021.675523)
Supplement: Supplementary file 1 [file Data_Sheet_1.docx]

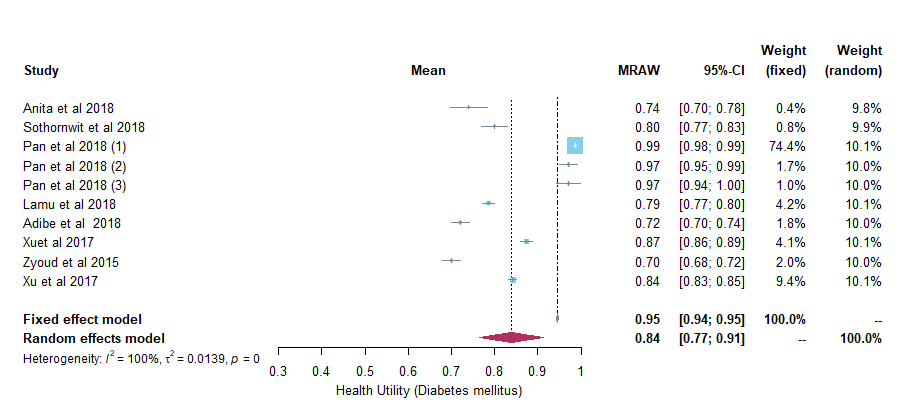


**Supplementary Figure 1a** Forest plot of the health utility of patients with diabetes mellitus.


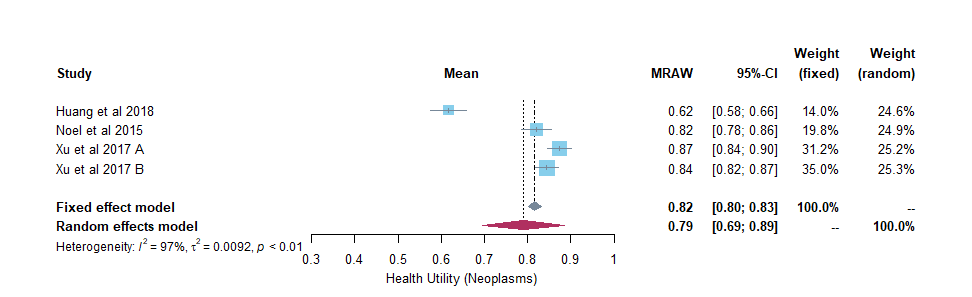


**Supplementary Figure 1b** Forest plot of the health utility of patients with neoplasms.


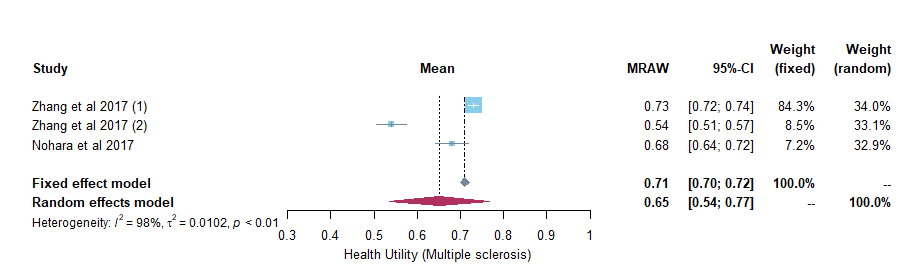


**Supplementary Figure 1c** Forest plot of the health utility of patients with multiple sclerosis


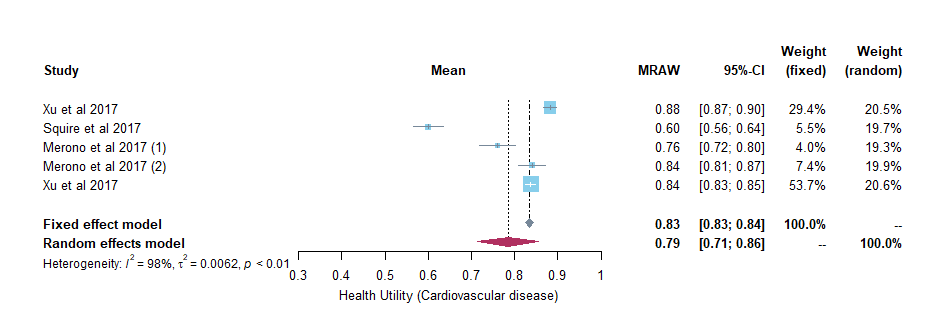


**Supplementary Figure 1d** Forest plot of the health utility of patients with cardiovascular diseases.


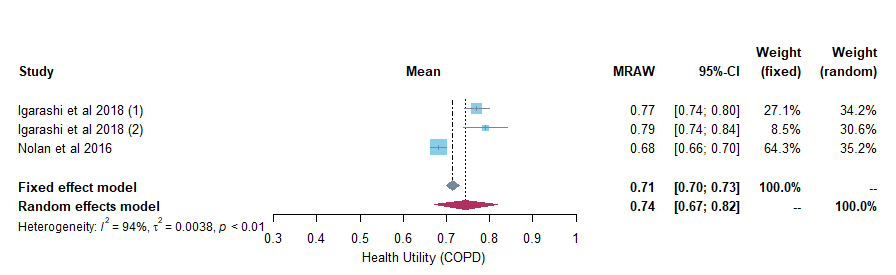


**Supplementary Figure 1e** Forest plot of the health utility of patients with chronic obstructive pneumonia disease.

*Chronic kidney disease and HIV infection are not presented in forest plots due to only one study left for each of the disease after removing utility values derived from crosswalk value set.
