# Supplementary material for: Health-Related Quality of Life in Patients With Different Diseases Measured With the EQ-5D-5L: A Systematic Review
Source: Front Public Health. 2021 Jun 29;9:675523. doi: 10.3389/fpubh.2021.675523 (PMC8275935; doi:10.3389/fpubh.2021.675523)
Supplement: Supplementary file 2 [file Table_1.DOCX]

**Supplementary Table 1** Literature search strategies and results

|  | **Search terms** | **Databases** | | | |
| --- | --- | --- | --- | --- | --- |
|  |  | Medline | Embase | Web of science | Cochrane library |
| #1 | quality of life | 325,138 | 563,983 | 484,723 | 109,498 |
| #2 | QoL | 194,529 | 69,654 | 35,665 | 18,955 |
| #3 | HRQoL | 15,399 | 25,378 | 15,460 | 5,079 |
| #4 | HRQL | 3,377 | 5,701 | 3,332 | 1,085 |
| #5 | #1-#4/OR | 326,685 | 569,585 | 486,839 | 112,623 |
| #6 | eq-5d | 7,432 | 14,561 | 8,292 | 6,137 |
| #7 | eq-5d-5l | 807 | 1,841 | 930 | 1,473 |
| #8 | five level | 787 | 1,012 | 261,844 | 20,363 |
| #9 | 5l | 1,496 | 2,910 | 3,619 | 1,625 |
| #10 | EuroQol | 5,184 | 8,316 | 5,700 | 3,669 |
| #11 | five dimension | 244 | 331 | 31,764 | 711 |
| #12 | #6-#11/OR | 11,134 | 20,244 | 299,820 | 27,947 |
| #13 | #5 and #12 | 8,428 | 15,852 | 15,705 | 12,885 |
| #14 | RCT | 21,194 | 36,800 | 21,407 | 30,179 |
| #15 | Randomized Controlled Trial | 520,448 | 763,167 | 405,488 | 956,636 |
| #16 | clinical trial | 693,889 | 1,511,577 | 641,990 | 927,931 |
| #17 | #14-#16/OR | 952,881 | 1,802,968 | 879,931 | 1,126,490 |
| #18 | #13 not #17 | 6,418 | 11,511 | 11,959 | 826 |
| #19 | disease | 4,192,679 | 7,913,824 | 4,095,895 | 364,056 |
| #20 | #18 and #19 | 1,649 | 5,079 | 2,736 | 36 |
|  | | | |  |  |
