# Supplementary material for: Health-Related Quality of Life in Patients With Different Diseases Measured With the EQ-5D-5L: A Systematic Review
Source: Front Public Health. 2021 Jun 29;9:675523. doi: 10.3389/fpubh.2021.675523 (PMC8275935; doi:10.3389/fpubh.2021.675523)
Supplement: Supplementary file 3 [file Table_2.DOCX]

**Supplementary Table 2** AHRQ checklist scores

|  | 1 | 2 | 3 | 4 | 5 | 6 | 7 | 8 | 9 | 10 | 11 | Scores |
| --- | --- | --- | --- | --- | --- | --- | --- | --- | --- | --- | --- | --- |
| Natasya et al 2018^[14]^ | Y | Y | N | U | N | Y | U | Y | U | Y | U | 5 |
| Sothornwit et al 2018^[15]^ | Y | Y | Y | Y | N | Y | N | Y | N | N | U | 6 |
| Pan et al 2018^[18]^ | Y | Y | N | Y | U | Y | N | Y | N | N | U | 5 |
| Lamu et al 2018^[19]^ | Y | Y | N | Y | Y | Y | N | Y | N | Y | U | 7 |
| Adibe et al 2018^[20]^ | Y | Y | U | U | U | U | U | Y | Y | Y | U | 5 |
| Arifin et al 2019^[21]^ | Y | Y | N | U | N | Y | Y | Y | N | Y | N | 6 |
| Schmitt et al 2018^[22]^ | Y | Y | Y | Y | N | N | Y | Y | N | Y | U | 7 |
| Collado et al 2015^[23]^ | Y | Y | Y | Y | N | N | N | N | Y | Y | U | 6 |
| Khatib et al 2018^[24]^ | Y | Y | Y | Y | N | Y | Y | Y | N | Y | U | 8 |
| Zyoud et al 2015^[25]^ | Y | Y | Y | Y | U | N | N | N | N | Y | U | 5 |
| Xu et al 2017^[26]^ | Y | Y | U | U | U | Y | Y | U | N | Y | U | 5 |
| Pan et al 2016^[27]^ | Y | Y | Y | Y | N | Y | Y | N | N | Y | U | 7 |
| Huang et al 2018^[28]^ | Y | Y | N | Y | N | Y | U | Y | Y | Y | U | 7 |
| Gavin et al 2016^[29]^ | Y | Y | N | U | Y | Y | Y | Y | N | Y | U | 7 |
| Lloyd et al 2015^[30]^ | Y | Y | N | Y | N | N | Y | N | N | Y | U | 5 |
| Philipp-Dormston et al 2018^[31]^ | Y | Y | Y | Y | N | N | Y | Y | Y | Y | U | 8 |
| Noel et al 2015^[32]^ | Y | Y | Y | Y | U | N | N | N | N | Y | U | 5 |
| Mastboom et al 2018^[33]^ | Y | Y | Y | Y | N | N | Y | N | N | Y | U | 6 |
| Zhang et al 2017^[34]^ | Y | Y | Y | N | N | N | Y | Y | Y | Y | U | 7 |
| Algahtani et al 2017^[35]^ | Y | Y | Y | Y | N | N | Y | Y | N | Y | U | 7 |
| Fogarty et al 2012^[36]^ | Y | U | U | U | N | Y | Y | Y | Y | Y | U | 6 |
| Carney et al 2018^[37]^ | Y | Y | Y | Y | N | Y | Y | N | N | Y | U | 7 |
| Nohara et al 2017^[38]^ | Y | Y | Y | Y | N | Y | Y | N | N | Y | U | 7 |
| Barin et al 2018^[39]^ | Y | Y | Y | Y | N | Y | Y | Y | N | Y | U | 8 |
| Buanes et al 2015^[40]^ | Y | Y | N | Y | U | N | Y | N | N | Y | U | 5 |
| Berg et al 2017^[41]^ | Y | Y | Y | Y | N | Y | Y | Y | N | Y | U | 9 |
| Squire et al 2017^[42]^ | Y | Y | Y | Y | N | N | N | Y | N | Y | U | 6 |
| Meroño et al 2017^[43]^ | Y | Y | Y | Y | N | Y | Y | Y | N | Y | Y | 9 |
| Tran et al 2018^[44]^ | Y | Y | N | U | U | Y | U | Y | U | Y | U | 5 |
| Wang et al 2018^[45]^ | Y | Y | N | Y | N | N | Y | N | N | Y | U | 5 |
| De Smedt et al 2016^[46]^ | Y | Y | Y | U | U | Y | N | N | N | Y | U | 5 |
| Garcia-Gordillo et al 2017^[47]^ | Y | Y | Y | Y | U | N | N | N | N | Y | U | 5 |
| Igarashi et al 2018^[48]^ | Y | Y | N | Y | Y | Y | N | Y | N | N | U | 6 |
| Lin et al 2014^[49]^ | Y | Y | Y | U | N | Y | U | N | Y | Y | N | 6 |
| Nolan et al 2016^[50]^ | Y | Y | Y | Y | N | N | Y | Y | N | Y | Y | 8 |
| Keaei et al 2016^[51]^ | Y | Y | Y | Y | N | Y | Y | N | N | Y | U | 7 |
| Dang et al 2018^[52]^ | Y | Y | Y | Y | N | N | Y | Y | N | Y | U | 7 |
| Tran et al 2012^[53]^ | Y | Y | N | U | N | Y | U | U | Y | Y | N | 6 |
| Van Duin et al 2017^[54]^ | Y | Y | Y | Y | N | N | N | Y | N | N | U | 5 |
| Yang et al 2015^[55]^ | Y | Y | Y | Y | N | Y | N | Y | N | N | U | 6 |
| Hiragi et al 2019^[56]^ | Y | Y | N | U | U | N | Y | U | N | Y | N | 4 |
| Zyoud et al 2016^[57]^ | Y | Y | Y | Y | N | Y | Y | Y | N | Y | U | 8 |
| Al-Jabi et al 2015^[58]^ | Y | Y | Y | Y | N | Y | Y | Y | N | Y | U | 8 |
| Van der Linde et al 2017^[59]^ | Y | Y | Y | Y | N | N | Y | Y | N | Y | U | 7 |
| Larsen et al 2015^[60]^ | Y | Y | Y | Y | U | N | Y | N | N | N | Y | 6 |
| Kim et al 2018^[61]^ | Y | Y | Y | N | N | N | Y | Y | N | Y | U | 6 |
| Chevreul et al 2016^[62]^ | Y | N | Y | U | U | N | Y | N | N | Y | U | 4 |
| Lopez-Bastida et al 2016^[63]^ | Y | Y | Y | N | N | N | Y | N | N | Y | U | 5 |
| Vaizey et al 2014^[64]^ | Y | Y | N | U | N | Y | U | Y | U | Y | N | 5 |
| Gibson et al 2014^[65]^ | Y | Y | N | U | N | Y | U | Y | U | Y | N | 5 |
| Yfantopoulos et al 2017^[66]^ | Y | Y | Y | Y | U | N | N | Y | N | N | U | 5 |
| Zhao et al 2017^[67]^ | Y | Y | Y | Y | Y | Y | N | Y | N | N | U | 7 |
| Choi et al 2018^[68]^ | Y | Y | Y | Y | U | N | N | Y | N | N | U | 5 |
| Chiowchanwisawaki et al 2019^[69]^ | Y | Y | N | U | N | Y | U | Y | U | Y | N | 5 |
| Alvarado-Bolanos et al 2015^[70]^ | Y | Y | N | Y | N | Y | N | N | N | N | U | 4 |
| Garcia-Gordillo et al 2014^[71]^ | Y | Y | Y | Y | N | N | Y | N | N | Y | U | 6 |
| Lee et al 2015^[72]^ | Y | Y | Y | Y | U | N | N | Y | N | Y | U | 6 |
| Lloyd et al 2017^[73]^ | Y | Y | Y | Y | N | N | Y | N | N | Y | U | 6 |
| Nordenfelt et al 2017^[74]^ | Y | Y | Y | Y | N | N | Y | N | N | Y | U | 6 |
| Nordenfelt et al 2014^[75]^ | Y | N | N | U | U | U | Y | N | N | Y | Y | 4 |
| Whitehurst et al 2016^[76]^ | Y | Y | N | Y | U | Y | Y | Y | N | Y | U | 8 |
| Engel et al 2018^[77]^ | Y | Y | Y | U | U | U | U | Y | N | N | U | 4 |
| Buckner et al 2017^[78]^ | Y | Y | Y | Y | N | Y | N | N | N | N | U | 5 |
| Kempton et al 2018^[79]^ | Y | Y | Y | Y | N | Y | Y | Y | N | N | U | 7 |
| Arraras et al 2018^[80]^ | Y | Y | Y | N | N | Y | Y | Y | N | Y | U | 7 |
| Kitic et al 2018^[81]^ | Y | Y | N | Y | N | N | N | Y | N | N | U | 4 |
| Tennvall et al 2015^[82]^ | Y | Y | Y | Y | N | N | Y | Y | N | Y | U | 7 |
| Gray et al 2018^[83]^ | Y | Y | N | U | U | Y | N | Y | N | N | U | 4 |
| Hernandez et al 2018^[84]^ | Y | Y | N | Y | Y | Y | Y | Y | N | Y | N | 8 |
| Wong et al 2018^[85]^ | Y | Y | Y | Y | N | N | Y | Y | N | Y | U | 7 |
| Cook et al 2019^[86]^ | Y | Y | N | U | N | Y | U | Y | U | Y | N | 5 |
| Van Dongen-Leunis et al 2016^[87]^ | Y | Y | Y | N | N | Y | N | Y | N | Y | N | 6 |
| Hendriksz et al 2014^[88]^ | Y | Y | N | U | N | Y | U | Y | U | Y | N | 5 |
| Andersson et al 2016^[89]^ | Y | Y | Y | Y | U | Y | Y | Y | N | Y | U | 8 |
| Mealy et al 2019^[90]^ | Y | Y | N | U | U | N | Y | Y | U | Y | N | 5 |
| Nikiphorou et al 2018^[91]^ | Y | Y | N | Y | U | N | N | Y | N | Y | U | 5 |
| Assche et al 2016^[92]^ | Y | Y | N | U | U | Y | Y | Y | N | Y | U | 6 |
| Mijnarends et al 2016^[93]^ | Y | Y | Y | Y | Y | N | N | Y | N | Y | U | 7 |
| Tran et al 2018^[94]^ | Y | Y | Y | Y | Y | Y | N | Y | N | N | U | 7 |
| Chevreul et al 2015^[95]^ | Y | Y | Y | Y | U | N | N | N | N | Y | U | 5 |
| Collado-Mateo et al 2017^[96]^ | Y | Y | Y | Y | U | N | N | Y | N | N | U | 5 |
| Chevreul et al 2015^[97]^ | Y | Y | Y | Y | U | N | N | Y | N | N | U | 5 |
| Juul-Kristensen et al 2017^[98]^ | Y | Y | Y | U | U | Y | Y | N | N | Y | U | 6 |
| Bewick et al 2018^[99]^ | Y | Y | Y | Y | N | N | Y | N | N | Y | U | 6 |
| Forestier-Zhang et al 2016^[100]^ | Y | Y | Y | Y | N | N | Y | N | N | Y | U | 6 |
| Katchamart et al 2019^[101]^ | Y | Y | N | U | U | N | Y | U | Y | Y | N | 5 |
| Román Ivorra et al 2019^[102]^ | Y | Y | N | U | N | Y | U | Y | U | Y | N | 5 |
| Aguirre et al 2016^[103]^ | N | Y | N | Y | U | Y | N | Y | N | Y | U | 5 |
| Wong et al 2017^[104]^ | Y | Y | Y | Y | N | Y | N | N | N | N | U | 5 |
| Christensen et al 2016^[105]^ | Y | Y | Y | U | U | N | Y | N | N | Y | U | 5 |
| Vo et al 2018^[106]^ | Y | Y | N | Y | N | N | Y | Y | N | Y | U | 6 |
| Voormolen et al 2019^[107]^ | Y | Y | N | U | N | Y | U | Y | U | Y | N | 5 |
| Lim et al 2017^[108]^ | Y | Y | Y | Y | N | Y | N | N | N | N | U | 5 |
| Villoro et al 2016^[109]^ | Y | Y | Y | Y | N | N | N | Y | Y | Y | U | 7 |
| Vermaire et al 2016^[110]^ | N | Y | Y | Y | U | N | Y | Y | N | Y | U | 6 |
| Lane et al 2017^[111]^ | Y | Y | Y | Y | N | Y | N | Y | N | N | U | 6 |
| Rencz et al 2018^[112]^ | Y | Y | Y | Y | N | Y | N | Y | Y | N | U | 7 |
| Chevreul et al 2015^[113]^ | Y | Y | Y | Y | N | N | Y | Y | N | Y | U | 7 |
| Y =yes. UC =unclear. N =no. AHRQ =Agency for Health Research and Quality. AHRQ checklists items: 1-Define the source of information. 2-List inclusion and exclusion criteria for exposed and unexposed subjects or refer to previous publications. 3-Indicate time period used for identifying patients. 4-Indicate whether or not subjects were consecutive if not population-based. 5-Indicate if evaluators of subjective components of study were masked to other aspects of the status of the participants. 6-Describe any assessments undertaken for quality assurance purposes. 7 Explain any patient exclusions from analysis. 8 Describe how confounding was assessed and/or controlled. 9-If applicable, explain how missing data were handled in the analysis. 10-Summarize patient response rates and completeness of data collection. 11-Clarify what follow up, if any, was expected and the percentage of patients for which incomplete data or follow-up was obtained. | | | | | | | | | | | | |
