# Supplementary material for: Health-Related Quality of Life in Patients With Different Diseases Measured With the EQ-5D-5L: A Systematic Review
Source: Front Public Health. 2021 Jun 29;9:675523. doi: 10.3389/fpubh.2021.675523 (PMC8275935; doi:10.3389/fpubh.2021.675523)
Supplement: Supplementary file 4 [file Table_3.DOCX]

| **Supplementary Table 3** Distribution of level 1-5 of each dimension in EQ-5D-5L | | | | | | | | | | | | | | | | | | | | | | | | | | |
| --- | --- | --- | --- | --- | --- | --- | --- | --- | --- | --- | --- | --- | --- | --- | --- | --- | --- | --- | --- | --- | --- | --- | --- | --- | --- | --- |
|  | **Diseases** | **Distribution of 5 dimensions** | | | | | | | | | | | | | | | | | | | | | | | | |
|  |  | Mobility (%) | | | | | Self-Care (%) | | | | | Usual Activities (%) | | | | | Pain/Discomfort (%) | | | | | Anxiety/Depression (%) | | | | |
|  |  | L1 | L2 | L3 | L4 | L5 | L1 | L2 | L3 | L4 | L5 | L1 | L2 | L3 | L4 | L5 | L1 | L2 | L3 | L4 | L5 | L1 | L2 | L3 | L4 | L5 |
| **Diabetes mellitus** |  |  |  |  |  |  |  |  |  |  |  |  |  |  |  |  |  |  |  |  |  |  |  |  |  |  |
| Natasya et al 2018^[14]^ | Diabetes mellitus (type 2) | 55.6 | 26.8 | 13.0 | 4.6 | 0.0 | 83.4 | 12.0 | 4.6 | 0.0 | 0.0 | 72.2 | 17.6 | 10.2 | 0.0 | 0.0 | 35.2 | 41.7 | 13.9 | 9.2 | 0.0 | 41.7 | 37.0 | 13.0 | 7.4 | 0.9 |
| Sothornwit et al 2018^[15]^ | Diabetes mellitus | - | - | - | - | - | - | - | - | - | - | - | - | - | - | - | - | - | - | - | - | - | - | - | - | - |
| Pan et al 2018^[18]^ | Diabetes without diabetic retinopathy | 92.9 | 7.1 | | | | 98.9 | 1.1 | | | | 99.3 | 0.7 | | | | 92.1 | 7.9 | | | | 96.8 | 3.2 | | | |
|  | Diabetes with unilateral retinopathy | 87.5 | 12.5 | | | | 94.6 | 5.4 | | | | 94.6 | 5.4 | | | | 83.9 | 16.1 | | | | 91.1 | 8.9 | | | |
|  | Diabetes with bilateral retinopathy | 92.2 | 7.8 | | | | 96.1 | 3.9 | | | | 94.1 | 5.9 | | | | 90.2 | 9.8 | | | | 94.1 | 5.9 | | | |
| Lamu et al 2018^[19]^ | Diabetes mellitus | - | - | - | - | - | - | - | - | - | - | - | - | - | - | - | - | - | - | - | - | - | - | - | - | - |
| Adibe et al 2018^[20]^ | Diabetes mellitus (type 2) | 38.8 | 29.9 | 26.5 | 2.7 | 2.0 | 68.0 | 20.4 | 5.4 | 2.0 | 4.1 | 37.4 | 32.7 | 19.0 | 6.8 | 4.1 | 17.0 | 38.1 | 42.2 | 2.7 | 0.0 | 28.6 | 60.5 | 10.9 | 0.0 | 0.0 |
| Arifn et al 2019^[21]^ | Diabetes mellitus (type 2) | 63.0 | 37.0 | | | | 88.0 | 12.0 | | | | 77.0 | 23.0 | | | | 39.0 | 61.0 | | | | 66.0 | 34.0 | | | |
| Schmitt et al 2018^[22]^ | Diabetes mellitus | - | - | - | - | - | - | - | - | - | - | - | - | - | - | - | - | - | - | - | - | - | - | - | - | - |
| Collado et al 2015^[23]^ | Diabetes mellitus | 53.2 | 16.7 | 15.8 | 11.0 | 3.3 | 76.4 | 8.6 | 6.7 | 4.6 | 3.7 | 62.5 | 14.8 | 11.0 | 5.3 | 6.3 | 45.6 | 22.3 | 20.1 | 10.4 | 1.3 | 70.6 | 14.5 | 9.5 | 3.9 | 0.8 |
| Khatib et al 2018^[24]^ | Diabetes mellitus (type 2) | - | - | - | - | - | - | - | - | - | - | - | - | - | - | - | - | - | - | - | - | - | - | - | - | - |
| Zyoud et al 2015^[25]^ | Diabetes mellitus (type 2) | - | - | - | - | - | - | - | - | - | - | - | - | - | - | - | - | - | - | - | - | - | - | - | - | - |
| Xu et al 2017^[26]^ | Diabetes mellitus | - | - | - | - | - | - | - | - | - | - | - | - | - | - | - | - | - | - | - | - | - | - | - | - | - |
| Pan et al 2016^[27]^ | Diabetes mellitus (type 2) | - | - | - | - | - | - | - | - | - | - | - | - | - | - | - | - | - | - | - | - | - | - | - | - | - |
| **Neoplasms** | | | | | | | | | | | | | | | | | | | | | | | | | | |
| Huang et al 2018^[28]^ | Colorectal cancer | 53.7 | 14.3 | 11.3 | 10.0 | 10.7 | 51.0 | 15.4 | 12.0 | 11.3 | 10.3 | 46.7 | 18.3 | 13.3 | 12.0 | 11.7 | 39.7 | 25.0 | 25.3 | 7.0 | 3.0 | 40.7 | 23.3 | 24.3 | 9.3 | 2.4 |
| Gavin et al 2016^[29]^ | Prostate cancer | - | - | - | - | - | - | - | - | - | - | - | - | - | - | - | - | - | - | - | - | - | - | - | - | - |
| Lloyd et al 2015^[30]^ | Prostate cancer | - | - | - | - | - | - | - | - | - | - | - | - | - | - | - | - | - | - | - | - | - | - | - | - | - |
| Philipp-Dormston et al 2018^[31]^ | Basal cell carcinoma | - | - | - | - | - | - | - | - | - | - | - | - | - | - | - | - | - | - | - | - | - | - | - | - | - |
|  | Squamous cell carcinoma | - | - | - | - | - | - | - | - | - | - | - | - | - | - | - | - | - | - | - | - | - | - | - | - | - |
| Noel et al 2015^[32]^ | Squamous cell carcinoma | - | - | - | - | - | - | - | - | - | - | - | - | - | - | - | - | - | - | - | - | - | - | - | - | - |
| Mastboom et al 2018^[33]^ | Diffuse tenosynovial giant cell tumor | - | - | - | - | - | - | - | - | - | - | - | - | - | - | - | - | - | - | - | - | - | - | - | - | - |
|  | Localized tenosynovial giant cell tumor | - | - | - | - | - | - | - | - | - | - | - | - | - | - | - | - | - | - | - | - | - | - | - | - | - |
| Xu et al 2017^[26]^ | Cancer | - | - | - | - | - | - | - | - | - | - | - | - | - | - | - | - | - | - | - | - | - | - | - | - | - |
| **Multiple sclerosis** |  |  |  |  |  |  |  |  |  |  |  |  |  |  |  |  |  |  |  |  |  |  |  |  |  |  |
| Zhang et al 2017^[34]^ | Relapse-onset multiple sclerosis | - | - | - | - | - | - | - | - | - | - | - | - | - | - | - | - | - | - | - | - | - | - | - | - | - |
|  | Progressive-onset multiple sclerosis | - | - | - | - | - | - | - | - | - | - | - | - | - | - | - | - | - | - | - | - | - | - | - | - | - |
| Algahtani et al 2017^[35]^ | Multiple sclerosis | 27.1 | 18.8 | 18.2 | 10.6 | 25.3 | 39.7 | 8.9 | 8.3 | 9.9 | 33.2 | 31.8 | 15.8 | 16.1 | 16.8 | 19.5 | 28.1 | 21.9 | 17.1 | 18.5 | 14.4 | 26.4 | 17.8 | 20.5 | 19.2 | 16.1 |
| Fogarty et al 2012^[36]^ | Multiple sclerosis | 29.9 | 24.8 | 20.6 | 14.0 | 10.7 | 63.8 | 16.4 | 9.9 | 2.8 | 7.0 | 29.4 | 21.0 | 27.6 | 12.6 | 9.3 | 32.7 | 30.4 | 24.3 | 10.3 | 2.3 | 45.8 | 33.6 | 16.4 | 4.2 | 0.0 |
| Carney et al 2018^[37]^ | Multiple sclerosis | - | - | - | - | - | - | - | - | - | - | - | - | - | - | - | - | - | - | - | - | - | - | - | - | - |
| Nohara et al 2017^[38]^ | Multiple sclerosis | - | - | - | - | - | - | - | - | - | - | - | - | - | - | - | - | - | - | - | - | - | - | - | - | - |
| Barin et al 2018^[39]^ | Multiple sclerosis | - | - | - | - | - | - | - | - | - | - | - | - | - | - | - | - | - | - | - | - | - | - | - | - | - |
| **Cardiovascular disease** |  |  |  |  |  |  |  |  |  |  |  |  |  |  |  |  |  |  |  |  |  |  |  |  |  |  |
| Buanes et al 2015^[40]^ | Cardiac arrest | - | - | - | - | - | - | - | - | - | - | - | - | - | - | - | - | - | - | - | - | - | - | - | - | - |
| Berg et al 2017^[41]^ | Heart disease | - | - | - | - | - | - | - | - | - | - | - | - | - | - | - | - | - | - | - | - | - | - | - | - | - |
| Squire et al 2017^[42]^ | Heart failure | - | - | - | - | - | - | - | - | - | - | - | - | - | - | - | - | - | - | - | - | - | - | - | - | - |
| Merono et al 2017^[43]^ | Iron deficiency in acute coronary syndrome | 48.0 | 52.0 | | | | 80.0 | 20.0 | | | | 51.0 | 49.0 | | | | 50.0 | 50.0 | | | | 39.0 | 61.0 | | | |
|  | Acute coronary syndrome non-iron deficiency | 71.0 | 29.0 | | | | 88.0 | 12.0 | | | | 67.0 | 33.0 | | | | 51.0 | 49.0 | | | | 48.0 | 52.0 | | | |
| Tran et al 2018^[44]^ | Cardiovascular disease | 75.2 | 24.8 | | | | 80.2 | 19.8 | | | | 77.3 | 22.7 | | | | 61.2 | 38.8 | | | | 64.8 | 35.2 | | | |
| Wang et al 2018^[45]^ | Atrial fibrillation | - | - | - | - | - | - | - | - | - | - | - | - | - | - | - | - | - | - | - | - | - | - | - | - | - |
| Xu et al 2017^[26]^ | Heart disease | - | - | - | - | - | - | - | - | - | - | - | - | - | - | - | - | - | - | - | - | - | - | - | - | - |
| De Smedt et al 2016^[46]^ | Stable coronary disease | - | - | - | - | - | - | - | - | - | - | - | - | - | - | - | - | - | - | - | - | - | - | - | - | - |
| **COPD** |  |  |  |  |  |  |  |  |  |  |  |  |  |  |  |  |  |  |  |  |  |  |  |  |  |  |
| Garcia-Gordillo et al 2017^[47]^ | COPD | 54.6 | 17.1 | 14.6 | 11.0 | 2.7 | 77.8 | 8.9 | 6.5 | 3.2 | 3.6 | 62.5 | 15.2 | 11.3 | 5.5 | 5.3 | 42.9 | 22.8 | 21.1 | 11.7 | 1.1 | 65.1 | 15.7 | 11.9 | 5.2 | 1.1 |
| Igarashi et al 2018^[48]^ | COPD age≥ 65years | 43.7 | 27.2 | 14.6 | 13.2 | 1.3 | 73.5 | 13.2 | 7.3 | 6.0 | 0.0 | 53.3 | 22.7 | 16.0 | 8.0 | 0.0 | 62.3 | 23.8 | 11.3 | 2.0 | 0.7 | 64.9 | 26.5 | 6.6 | 2.0 | 0.0 |
|  | COPD age <65 years | 56.3 | 22.5 | 11.3 | 7.0 | 2.8 | 76.1 | 11.3 | 7.0 | 4.2 | 1.4 | 56.3 | 26.8 | 7.0 | 9.9 | 0.0 | 70.0 | 20.0 | 4.3 | 4.3 | 1.4 | 61.4 | 24.3 | 7.1 | 5.7 | 1.4 |
| Lin et al 2014^[49]^ | COPD | 36.4 | 26.7 | 28.2 | 7.8 | 0.9 | 80.5 | 13.1 | 5.1 | 0.8 | 0.6 | 45.2 | 27.3 | 20.2 | 4.8 | 2.5 | 38.1 | 30.9 | 23.9 | 6.4 | 0.8 | 63.7 | 20.8 | 11.8 | 3.0 | 0.8 |
| Nolan et al 2016^[50]^ | COPD | - | - | - | - | - | - | - | - | - | - | - | - | - | - | - | - | - | - | - | - | - | - | - | - | - |
| **HIV infection** |  |  |  |  |  |  |  |  |  |  |  |  |  |  |  |  |  |  |  |  |  |  |  |  |  |  |
| Keaei et al 2016^[51]^ | HIV/AIDS | 81.2 | 8.0 | 6.5 | 5.1 | 0.7 | 91.3 | 5.1 | 2.9 | 3.6 | 1.5 | 84.1 | 9.4 | 5.8 | 0.0 | 0.7 | 61.6 | 26.8 | 5.8 | 3.6 | 4.4 | 59.4 | 24.6 | 10.9 | 5.1 | 2.2 |
| Dang et al 2018^[52]^ | HIV-positive | 79.5 | 20.5 | | | | 90.3 | 9.7 | | | | 83.4 | 16.6 | | | | 62.3 | 37.7 | | | | 55.1 | 44.9 | | | |
| Tran et al 2012^[53]^ | HIV | 54.9 | 27.6 | 9.1 | 5.2 | 3.3 | 79.8 | 11.5 | 4.0 | 2.6 | 2.1 | 64.6 | 21.8 | 6.4 | 3.9 | 3.4 | 41.8 | 42.8 | 9.4 | 3.6 | 2.4 | 27.5 | 42.5 | 16.9 | 8.6 | 4.5 |
| Van Duin et al 2017^[54]^ | HIV | - | - | - | - | - | - | - | - | - | - | - | - | - | - | - | - | - | - | - | - | - | - | - | - | - |
| **Chronic kidney disease** |  |  |  |  |  |  |  |  |  |  |  |  |  |  |  |  |  |  |  |  |  |  |  |  |  |  |
| Yang et al 2015^[55]^ | End-stage renal disease | - | - | - | - | - | - | - | - | - | - | - | - | - | - | - | - | - | - | - | - | - | - | - | - | - |
| Hiragi et al 2019^[56]^ | Chronic kidney disease | - | - | - | - | - | - | - | - | - | - | - | - | - | - | - | - | - | - | - | - | - | - | - | - | - |
| Zyoud et al 2016^[57]^ | End-stage renal disease | 72.7 | 27.3 | | | | 45.3 | 54.7 | | | | 62.5 | 37.5 | | | | 74.5 | 25.5 | | | | 64.8 | 35.2 | | | |
| **Hypertension** |  |  |  |  |  |  |  |  |  |  |  |  |  |  |  |  |  |  |  |  |  |  |  |  |  |  |
| Al-Jabi et al 2015^[58]^ | Hypertension | - | - | - | - | - | - | - | - | - | - | - | - | - | - | - | - | - | - | - | - | - | - | - | - | - |
| Xu et al 2017^[26]^ | Hypertension | - | - | - | - | - | - | - | - | - | - | - | - | - | - | - | - | - | - | - | - | - | - | - | - | - |
| **Fractures** |  |  |  |  |  |  |  |  |  |  |  |  |  |  |  |  |  |  |  |  |  |  |  |  |  |  |
| Van der Linde et al 2017^[59]^ | Midshaft clavicular fractures | - | - | - | - | - | - | - | - | - | - | - | - | - | - | - | - | - | - | - | - | - | - | - | - | - |
| Larsen et al 2015^[60]^ | Femoral shaft fracture | - | - | - | - | - | - | - | - | - | - | - | - | - | - | - | - | - | - | - | - | - | - | - | - | - |
| Kim et al 2018^[61]^ | Osteoporotic vertebral compression fracture | - | - | - | - | - | - | - | - | - | - | - | - | - | - | - | - | - | - | - | - | - | - | - | - | - |
| **Prader–Willi syndrome** |  |  |  |  |  |  |  |  |  |  |  |  |  |  |  |  |  |  |  |  |  |  |  |  |  |  |
| Chevreul et al 2016^[62]^ | Prader–Willi syndrome | - | - | - | - | - | - | - | - | - | - | - | - | - | - | - | - | - | - | - | - | - | - | - | - | - |
| Lopez-Bastida et al 2016^[63]^ | Prader-Willi syndrome | - | - | - | - | - | - | - | - | - | - | - | - | - | - | - | - | - | - | - | - | - | - | - | - | - |
| **Ulcerative colitis** |  |  |  |  |  |  |  |  |  |  |  |  |  |  |  |  |  |  |  |  |  |  |  |  |  |  |
| Vaizey et al 2014^[64]^ | Ulcerative colitis remission | - | - | - | - | - | - | - | - | - | - | - | - | - | - | - | - | - | - | - | - | - | - | - | - | - |
| Gibson et al 2014^[65]^ | Ulcerative colitis remission | - | - | - | - | - | - | - | - | - | - | - | - | - | - | - | - | - | - | - | - | - | - | - | - | - |
| Vaizey et al 2014^[64]^ | Ulcerative colitis | - | - | - | - | - | - | - | - | - | - | - | - | - | - | - | - | - | - | - | - | - | - | - | - | - |
| Gibson et al 2014^[65]^ | Ulcerative colitis | - | - | - | - | - | - | - | - | - | - | - | - | - | - | - | - | - | - | - | - | - | - | - | - | - |
| Vaizey et al 2014^[64]^ | Ulcerative colitis mild | - | - | - | - | - | - | - | - | - | - | - | - | - | - | - | - | - | - | - | - | - | - | - | - | - |
| Gibson et al 2014^[65]^ | Ulcerative colitis mild | - | - | - | - | - | - | - | - | - | - | - | - | - | - | - | - | - | - | - | - | - | - | - | - | - |
| **Psoriasis** |  |  |  |  |  |  |  |  |  |  |  |  |  |  |  |  |  |  |  |  |  |  |  |  |  |  |
| Yfantopoulos et al 2017^[66]^ | Psoriasis | 81.6 | 18.4 | | | | 90.2 | 9.8 | | | | 84.3 | 15.7 | | | | 66.4 | 33.6 | | | | 22.0 | 78.0 | | | |
| Zhao et al 2017^[67]^ | Psoriasis | - | - | - | - | - | - | - | - | - | - | - | - | - | - | - | - | - | - | - | - | - | - | - | - | - |
| **Ankylosing spondylitis** | | | | | | | | | | | | | | | | | | | | | | | | | | |
| Choi et al 2018^[68]^ | Ankylosing spondylitis | - | - | - | - | - | - | - | - | - | - | - | - | - | - | - | - | - | - | - | - | - | - | - | - | - |
| Chiowchanwisawakit et al 2019^[69]^ | Ankylosing spondylitis | 22.7 | 42.9 | 26.1 | 7.6 | 0.8 | 63.0 | 20.2 | 16.0 | 0.0 | 0.8 | 31.1 | 38.7 | 25.2 | 4.2 | 0.8 | 6.7 | 47.1 | 34.5 | 10.1 | 1.7 | 45.4 | 37.0 | 12.6 | 4.2 | 0.8 |
| **Actinic keratosis** |  |  |  |  |  |  |  |  |  |  |  |  |  |  |  |  |  |  |  |  |  |  |  |  |  |  |
| Tennvall et al 2015^[82]^ | Actinic keratosis | 79.0 | 11.0 | 6.0 | 4.0 | 0.0 | 93.0 | 4.0 | 2.0 | 1.0 | 0.0 | 82.0 | 12.0 | 4.0 | 1.0 | 1.0 | 61.0 | 27.0 | 10.0 | 1.0 | 1.0 | 78.0 | 18.0 | 3.0 | 1.0 | 0.0 |
| Philipp-Dormston et al 2018^[31]^ | Actinic keratosis | - | - | - | - | - | - | - | - | - | - | - | - | - | - | - | - | - | - | - | - | - | - | - | - | - |
| **Parkinson's disease** |  |  |  |  |  |  |  |  |  |  |  |  |  |  |  |  |  |  |  |  |  |  |  |  |  |  |
| Alvarado-Bolanos et al 2015^[70]^ | Parkinson's disease | - | - | - | - | - | - | - | - | - | - | - | - | - | - | - | - | - | - | - | - | - | - | - | - | - |
| Garcia-Gordillo et al 2014^[71]^ | Parkinson’s disease | 24.1 | 34.6 | 28.6 | 12.8 | 0.0 | 39.8 | 33.8 | 13.5 | 10.5 | 2.3 | 24.1 | 36.1 | 25.6 | 11.3 | 3.0 | 24.1 | 30.1 | 33.1 | 7.5 | 4.5 | 33.8 | 35.3 | 27.8 | 0.0 | 3.0 |
| **Overactive bladder** |  |  |  |  |  |  |  |  |  |  |  |  |  |  |  |  |  |  |  |  |  |  |  |  |  |  |
| Lee et al 2015^[72]^ | Overactive bladder | - | - | - | - | - | - | - | - | - | - | - | - | - | - | - | - | - | - | - | - | - | - | - | - | - |
| Lloyd et al 2017^[73]^ | Idiopathic overactive bladder | - | - | - | - | - | - | - | - | - | - | - | - | - | - | - | - | - | - | - | - | - | - | - | - | - |
| **Hereditary angioedema** |  |  |  |  |  |  |  |  |  |  |  |  |  |  |  |  |  |  |  |  |  |  |  |  |  |  |
| Nordenfelt et al 2017^[74]^ | Hereditary angioedema | - | - | - | - | - | - | - | - | - | - | - | - | - | - | - | - | - | - | - | - | - | - | - | - | - |
| Nordenfelt et al 2014^[75]^ | Hereditary angioedema | - | - | - | - | - | - | - | - | - | - | - | - | - | - | - | - | - | - | - | - | - | - | - | - | - |
| **Spinal cord injury** |  |  |  |  |  |  |  |  |  |  |  |  |  |  |  |  |  |  |  |  |  |  |  |  |  |  |
| Whitehurst et al 2016^[76]^ | Spinal cord injury | 3.0 | 4.0 | 10.0 | 12.0 | 72.0 | 33.0 | 24.0 | 16.0 | 7.0 | 20.0 | 20.0 | 28.0 | 32.0 | 13.0 | 7.0 | 8.0 | 33.0 | 35.0 | 19.0 | 4.0 | 43.0 | 33.0 | 18.0 | 5.0 | 1.0 |
| Engel et al 2018^[77]^ | Spinal cord injury | - | - | - | - | - | - | - | - | - | - | - | - | - | - | - | - | - | - | - | - | - | - | - | - | - |
| **Schizophrenia** |  |  |  |  |  |  |  |  |  |  |  |  |  |  |  |  |  |  |  |  |  |  |  |  |  |  |
| Arraras et al 2018^[80]^ | Schizophrenia and schizoaffective disorder | - | - | - | - | - | - | - | - | - | - | - | - | - | - | - | - | - | - | - | - | - | - | - | - | - |
| Kitic et al 2018^[81]^ | Schizophrenia | - | - | - | - | - | - | - | - | - | - | - | - | - | - | - | - | - | - | - | - | - | - | - | - | - |
| **Hemophilia** |  |  |  |  |  |  |  |  |  |  |  |  |  |  |  |  |  |  |  |  |  |  |  |  |  |  |
| Buckner et al 2017^[78]^ | Hemophilia B | 22.0 | 58.0 | 16.0 | 3.0 | 1.0 | 25.0 | 56.0 | 14.0 | 6.0 | 0.0 | 13.0 | 46.0 | 27.0 | 14.0 | 0.0 | 7.0 | 46.0 | 36.0 | 11.0 | 0.0 | 19.0 | 40.0 | 31.0 | 10.0 | 0.0 |
| Kempton et al 2018^[79]^ | Hemophilia | 38.6 | 29.7 | 22.3 | 7.6 | 1.8 | 81.1 | 14.2 | 3.9 | 0.8 | 0.0 | 46.8 | 31.8 | 15.3 | 3.9 | 2.2 | 23.9 | 33.2 | 28.2 | 12.4 | 2.3 | 56.6 | 26.2 | 11.6 | 3.7 | 1.9 |
| **Asthma** |  |  |  |  |  |  |  |  |  |  |  |  |  |  |  |  |  |  |  |  |  |  |  |  |  |  |
| Gray et al 2018^[83]^ | Asthma | - | - | - | - | - | - | - | - | - | - | - | - | - | - | - | - | - | - | - | - | - | - | - | - | - |
| Hernandez et al 2018^[84]^ | Asthma | - | - | - | - | - | - | - | - | - | - | - | - | - | - | - | - | - | - | - | - | - | - | - | - | - |
| **Hepatitis** |  |  |  |  |  |  |  |  |  |  |  |  |  |  |  |  |  |  |  |  |  |  |  |  |  |  |
| Wong et al 2018^[85]^ | Autoimmune Hepatitis | - | - | - | - | - | - | - | - | - | - | - | - | - | - | - | - | - | - | - | - | - | - | - | - | - |
| Cook et al 2019^[86]^ | Non-alcoholic steatohepatitis | - | - | - | - | - | - | - | - | - | - | - | - | - | - | - | - | - | - | - | - | - | - | - | - | - |
| **Other diseases** |  |  |  |  |  |  |  |  |  |  |  |  |  |  |  |  |  |  |  |  |  |  |  |  |  |  |
| Van Dongen-Leunis et al 2016^[87]^ | Acute leukemia | - | - | - | - | - | - | - | - | - | - | - | - | - | - | - | - | - | - | - | - | - | - | - | - | - |
|  | Acute leukemia | - | - | - | - | - | - | - | - | - | - | - | - | - | - | - | - | - | - | - | - | - | - | - | - | - |
| Hendriksz et al 2014^[88]^ | Morquio A syndrome | - | - | - | - | - | - | - | - | - | - | - | - | - | - | - | - | - | - | - | - | - | - | - | - | - |
| Andersson et al 2016^[89]^ | Nocturia | - | - | - | - | - | - | - | - | - | - | - | - | - | - | - | - | - | - | - | - | - | - | - | - | - |
| Mealy et al 2019^[90]^ | Neuromyelitis optica spectrum disorder | 33.3 | 66.7 | | | | 66.7 | 33.3 | | | | 38.1 | 61.9 | | | | 23.8 | 76.2 | | | | 28.6 | 71.4 | | | |
| Nikiphorou et al 2018^[91]^ | Spondyloarthritis | - | - | - | - | - | - | - | - | - | - | - | - | - | - | - | - | - | - | - | - | - | - | - | - | - |
| Van Assche et al 2016^[92]^ | Ulcerative colitis | - | - | - | - | - | - | - | - | - | - | - | - | - | - | - | - | - | - | - | - | - | - | - | - | - |
| Mijnarends et al 2016^[93]^ | Sarcopenia | - | - | - | - | - | - | - | - | - | - | - | - | - | - | - | - | - | - | - | - | - | - | - | - | - |
| Tran et al 2018^[94]^ | Dengue fever | 37.7 | 62.3 | | | | 28.2 | 71.8 | | | | 35.4 | 64.6 | | | | 67.7 | 32.3 | | | | 35.9 | 64.1 | | | |
| Chevreul et al 2015^[95]^ | Cystic fibrosis | - | - | - | - | - | - | - | - | - | - | - | - | - | - | - | - | - | - | - | - | - | - | - | - | - |
| Collado-Mateo et al 2017^[96]^ | Fibromyalgia | - | - | - | - | - | - | - | - | - | - | - | - | - | - | - | - | - | - | - | - | - | - | - | - | - |
| Chevreul et al 2015^[97]^ | Fragile X syndrome | - | - | - | - | - | - | - | - | - | - | - | - | - | - | - | - | - | - | - | - | - | - | - | - | - |
| Juul-Kristensen et al 2017^[98]^ | Generalised joint hypermobility | - | - | - | - | - | - | - | - | - | - | - | - | - | - | - | - | - | - | - | - | - | - | - | - | - |
| Bewick et al 2018^[99]^ | Rhinosinusitis | 69.2 | 30.8 | | | | 90.4 | 9.6 | | | | 60.5 | 39.5 | | | | 32.7 | 67.3 | | | | 57.7 | 42.3 | | | |
| Forestier-Zhang et al 2016^[100]^ | Fibrous dysplasia | 43.0 | 21.0 | 19.0 | 17.0 | 0.0 | 62.0 | 26.0 | 10.0 | 3.0 | 0.0 | 33.0 | 31.0 | 10.0 | 19.0 | 7.0 | 2.0 | 31.0 | 36.0 | 21.0 | 10.0 | 38.0 | 29.0 | 24.0 | 10.0 | 0.0 |
|  | X-linked hypophosphatemia | 13.0 | 42.0 | 25.0 | 8.0 | 13.0 | 50.0 | 29.0 | 4.0 | 8.0 | 8.0 | 25.0 | 42.0 | 21.0 | 13.0 | 0.0 | 8.0 | 25.0 | 42.0 | 21.0 | 4.0 | 42.0 | 54.0 | 4.0 | 0.0 | 0.0 |
|  | Osteogenesis imperfecta | 19.0 | 33.0 | 23.0 | 14.0 | 12.0 | 61.0 | 23.0 | 7.0 | 5.0 | 5.0 | 35.0 | 30.0 | 19.0 | 12.0 | 5.0 | 7.0 | 33.0 | 44.0 | 9.0 | 7.0 | 40.0 | 40.0 | 14.0 | 7.0 | 0.0 |
| Katchamart et al 2019^[101]^ | Rheumatoid arthritis | 48.5 | 32.8 | 13.8 | 4.3 | 0.6 | 83.2 | 11.6 | 4.1 | 0.6 | 0.4 | 64.7 | 25.2 | 8.2 | 1.7 | 0.2 | 29.5 | 51.3 | 17.7 | 1.1 | 0.4 | 61.2 | 32.1 | 5.4 | 1.1 | 0.2 |
| Román Ivorral et al 2019^[102]^ | Systemic lupus erythematosus | - | - | - | - | - | - | - | - | - | - | - | - | - | - | - | - | - | - | - | - | - | - | - | - | - |
| Aguirre et al 2016^[103]^ | Dementia | - | - | - | - | - | - | - | - | - | - | - | - | - | - | - | - | - | - | - | - | - | - | - | - | - |
| Wong et al 2017^[104]^ | Adolescent idiopathic scoliosis | - | - | - | - | - | - | - | - | - | - | - | - | - | - | - | - | - | - | - | - | - | - | - | - | - |
| Christensen et al 2016^[105]^ | Opioid-induced constipation | - | - | - | - | - | - | - | - | - | - | - | - | - | - | - | - | - | - | - | - | - | - | - | - | - |
| Vo et al 2018^[106]^ | Migraine | - | - | - | - | - | - | - | - | - | - | - | - | - | - | - | - | - | - | - | - | - | - | - | - | - |
| Voormolen et al 2019^[107]^ | Post-concussion syndrome | - | - | - | - | - | - | - | - | - | - | - | - | - | - | - | - | - | - | - | - | - | - | - | - | - |
| Lim et al 2017^[108]^ | Stoma | - | - | - | - | - | - | - | - | - | - | - | - | - | - | - | - | - | - | - | - | - | - | - | - | - |
| Villoro et al 2016^[109]^ | Chronic depression | 69.9 | 12.7 | 10.4 | 5.3 | 1.6 | 86.7 | 4.8 | 5.3 | 1.6 | 1.7 | 71.4 | 10.9 | 10.8 | 4.2 | 2.8 | 42.6 | 20.8 | 24.2 | 11.0 | 1.4 | 26.5 | 27.1 | 26.6 | 16.5 | 3.3 |
| Vermaire et al 2016^[110]^ | Severe dental anxiety | - | - | - | - | - | - | - | - | - | - | - | - | - | - | - | - | - | - | - | - | - | - | - | - | - |
| Lane et al 2017^[111]^ | Symptomatic varicose vein | - | - | - | - | - | - | - | - | - | - | - | - | - | - | - | - | - | - | - | - | - | - | - | - | - |
| Rencz et al 2018^[112]^ | Crohn’s disease | 95.2 | 4.8 | 0.0 | 0.0 | 0.0 | 98.5 | 1.5 | 0.0 | 0.0 | 0.0 | 88.4 | 10.9 | 0.7 | 0.0 | 0.0 | 82.2 | 14.4 | 3.3 | 0.0 | 0.0 | 94.1 | 4.2 | 1.7 | 0.0 | 0.0 |
| Chevreul et al 2015^[113]^ | Systemic sclerosis | - | - | - | - | - | - | - | - | - | - | - | - | - | - | - | - | - | - | - | - | - | - | - | - | - |
| EQ-5D-5L, 5 level version of EuroQol 5-Dimensions; L, Level; RoI, the Republic of Ireland; NI, Northern Ireland; COPD, chronic obstructive pulmonary disease; HIV, human immunodeficiency virus; AIDS, acquired immunodeficiency syndrome. | | | | | | | | | | | | | | | | | | | | | | | | | | |
